# Deciphering chloramphenicol biotransformation mechanisms and microbial interactions via integrated multi-omics and cultivation-dependent approaches

**DOI:** 10.1186/s40168-022-01361-5

**Published:** 2022-10-24

**Authors:** Jiayu Zhang, Xiaoyan Li, Uli Klümper, Huaxin Lei, Thomas U. Berendonk, Fangliang Guo, Ke Yu, Chao Yang, Bing Li

**Affiliations:** 1 State Environmental Protection Key Laboratory of Microorganism Application and Risk Control, Tsinghua Shenzhen International Graduate School, Tsinghua University, Shenzhen, China; 2 School of Environment, Tsinghua University, Beijing, China; 3 Institute of Hydrobiology, Technische Universität Dresden, Dresden, Germany; 4 School of Environment and Energy, Shenzhen Graduate School, Peking University, Shenzhen, China; 5 Key Laboratory of Molecular Microbiology and Technology for Ministry of Education, College of Life Sciences, Nankai University, Tianjin, China

**Keywords:** Chloramphenicol biotransformation, Multi-omics, Genomics, Metagenomics, Metatranscriptomics, Proteomics, Interspecies interaction

## Abstract

**Background:**

As a widely used broad-spectrum antibiotic, chloramphenicol is prone to be released into environments, thus resulting in the disturbance of ecosystem stability as well as the emergence of antibiotic resistance genes. Microbes play a vital role in the decomposition of chloramphenicol in the environment, and the biotransformation processes are especially dependent on synergistic interactions and metabolite exchanges among microbes. Herein, the comprehensive chloramphenicol biotransformation pathway, key metabolic enzymes, and interspecies interactions in an activated sludge-enriched consortium were elucidated using integrated multi-omics and cultivation-based approaches.

**Results:**

The initial biotransformation steps were the oxidization at the C_1_-OH and C_3_-OH groups, the isomerization at C_2_, and the acetylation at C_3_-OH of chloramphenicol. Among them, the isomerization is an entirely new biotransformation pathway of chloramphenicol discovered for the first time. Furthermore, we identified a novel glucose-methanol-choline oxidoreductase responsible for the oxidization of the C_3_-OH group in *Sphingomonas* sp. and *Caballeronia* sp. Moreover, the subsequent biotransformation steps, corresponding catalyzing enzymes, and the microbial players responsible for each step were deciphered. Synergistic interactions between *Sphingomonas* sp. and *Caballeronia* sp. or *Cupriavidus* sp. significantly promoted chloramphenicol mineralization, and the substrate exchange interaction network occurred actively among key microbes.

**Conclusion:**

This study provides desirable strain and enzyme resources for enhanced bioremediation of chloramphenicol-contaminated hotspot sites such as pharmaceutical wastewater and livestock and poultry wastewater. The in-depth understanding of the chloramphenicol biotransformation mechanisms and microbial interactions will not only guide the bioremediation of organic pollutants but also provide valuable knowledge for environmental microbiology and biotechnological exploitation.

Video Abstract

**Supplementary Information:**

The online version contains supplementary material available at 10.1186/s40168-022-01361-5.

## Background

Chloramphenicol (CAP) is a broad-spectrum antibiotic and has been widely used in the medical treatment of bacterial infections. Despite a number of proven side effects, including aplastic anemia, marrow aplasia, visual impairment, and deafness in humans, it is still widely used in developing countries because of its low cost and excellent antibacterial properties against both gram-negative and gram-positive bacterial pathogens [1]. For example, 1230 tons of CAP were estimated to be consumed annually in China [2]. Due to its consistent use in human medical treatment, livestock and poultry breeding, and aquaculture, highly persistent CAP residues are continuously released into the environment and accumulate in water and soils, thus resulting in the disturbance of ecosystem stability as well as the emergence and spread of antibiotic resistance genes in the environment [3, 4]. It was reported that antibiotic concentrations ranged from ng/L to mg/L levels in aquatic environments worldwide [5], especially in pharmaceutical wastewater, which could reach up to 115 mg/L [6]. CAP concentration was as high as 3.1 mg/L in wastewater from an antibiotic pharmaceutical enterprise in China [7].

Microbes play a vital role in the decomposition of CAP in natural as well as engineered environments such as biological treatment systems for pharmaceutical wastewater treatment [8, 9]. Several studies investigated the metabolic pathways and mechanisms of CAP but mainly focused on the first biotransformation step. The nitroreduction, hydroxyl acetylation, and amide bond hydrolysis were the most common CAP-degrading processes in bacteria, and the corresponding enzymes were confirmed to be nitroreductase NfsB, CAP acetyltransferase CAT, and esterase estDL136 [10–12]. Still, a comprehensive understanding of the metabolic pathways, mechanisms, and corresponding enzymes is lacking. These are crucial for the exploitation in bioremediation applications. Moreover, previous studies mainly focused on investigating CAP biotransformation by pure cultures exclusively [10, 13, 14]. The missing step is understanding CAP biotransformation by microbial consortia, which is especially relevant for full-scale wastewater treatment plants where many processes such as aerobic biodegradation and anaerobic digestion rely on microbial interactions rather than the performance of single strains or species. Since biotransformation allows bacteria to resist CAP-induced bacteriostasis [10], comprehensive deciphering of the CAP biotransformation mechanisms will also provide new insights into CAP resistance or tolerance mechanisms.

The biotransformation of organics usually relies on the synergistic microbial metabolism to achieve desirable performance outcomes in engineered or natural environments [15, 16]. For instance, *Pseudomonas* sp. and *Pusillimonas* sp. could utilize products of bisphenol A produced by *Sphingonomas* spp. to enhance bisphenol A mineralization in a microbial consortium [17]. Deng et al. reported a partnership on sulfadiazine biodegradation between *Arthrobacter* sp. and *Pimelobacter* sp. in a sulfadiazine-degrading bioreactor [18]. They found *Pimelobacter* sp. could subsist on 2-aminopyrimidine converted from sulfadiazine by *Arthrobacter* sp. The unveiling of microbial interaction will be beneficial for developing appropriate bioremediation strategies.

Here, we took advantage of a microbial consortium (named CL) which was enriched from an activated sludge bioreactor with CAP as the sole carbon source and continuously passaged for 1.5 years. Its bacterial community structure and CAP biodegradation characteristics were primarily investigated via 16S rRNA amplicon sequencing and biodegradation batch tests [19]. Nevertheless, comprehensive insights into the metabolic mechanisms and microbial interactions during the biotransformation processes were still missing. In the current study, the detailed CAP biotransformation pathways, the key metabolic enzymes involved, and the interspecies interactions of members in this consortium were comprehensively elucidated using an integrated multi-omics (i.e., metagenomics, metatranscriptomics, and proteomics) and cultivation-based approach. An in-depth understanding of the CAP biotransformation mechanisms and microbial interactions will not only guide the bioremediation of organic pollutants but also provide a valuable knowledge base for environmental microbiology and biotechnological applications.

## Results and discussion

### Consortium CL exhibits efficient CAP biotransformation performance

**Fig. 1:**
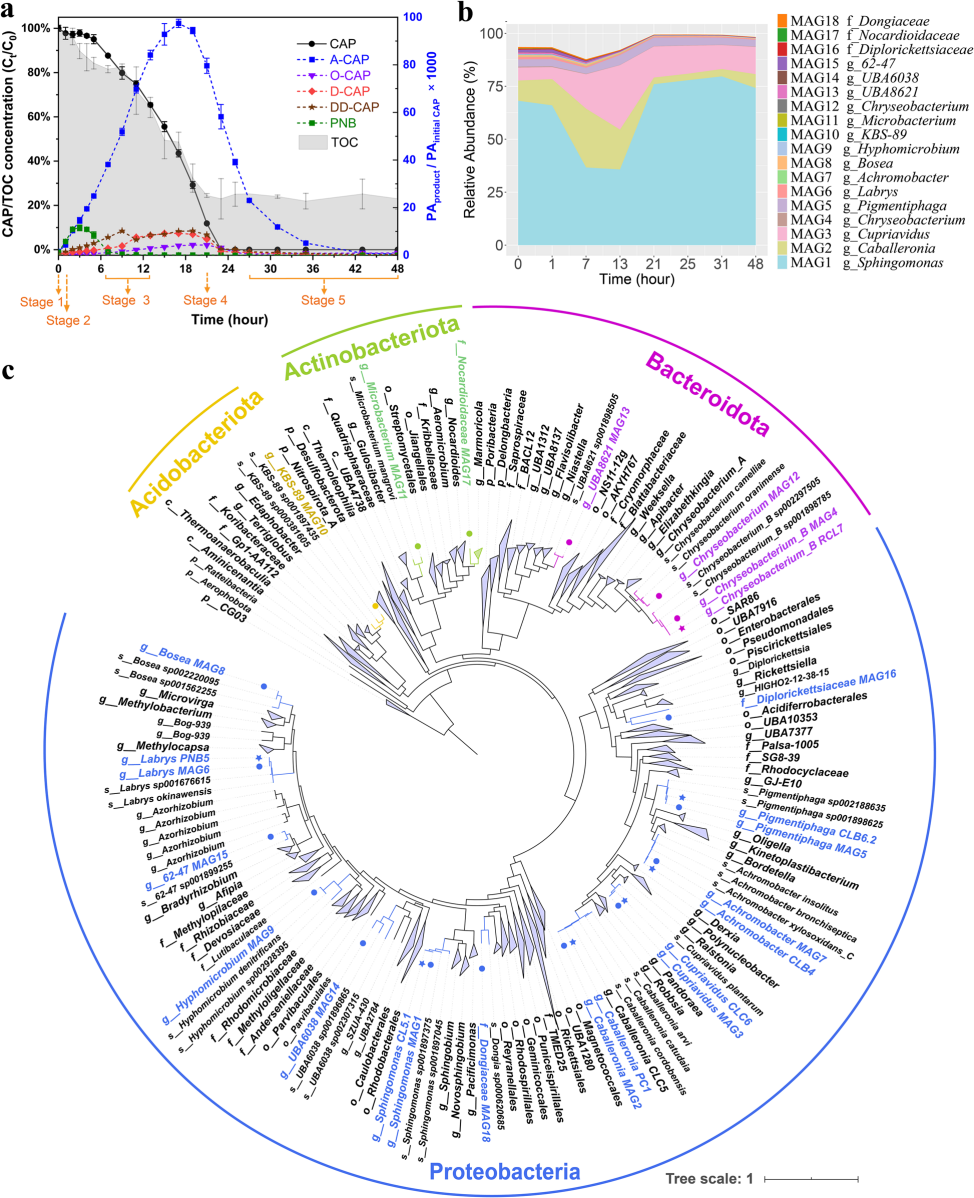
The bacterial community structure of CAP-degrading consortium CL. **a** The biotransformation of CAP by consortium CL. CAP metabolites were semi-quantified according to the proportion of their peak areas (PA) to the peak area of CAP at the initial concentration of 120 mg/L. Data are presented as mean values ± SD (*n* = 3). **b** The relative abundances of 18 MAGs recovered from metagenomic data. Data are presented as mean values of triplicates. **c** Phylogenetic tree of seven isolated strain genomes and 18 reconstructed MAGs relative to 2170 reference genomes based on 120 single-copy marker proteins for bacteria

The microbial consortium CL possesses the ability to utilize CAP as the sole carbon and energy source. It was initially enriched from an activated sludge bioreactor in our lab. During CAP-degrading batch experiments, the microbiome of consortium CL was sampled for metagenomic sequencing and metatranscriptomic sequencing at 0 (before CAP dosing, defined as stage 1), 1, 7, 13, 21, 25, 31, and 48 h after CAP dosing at an initial concentration of 120 mg/L. This covered all the stages of CAP biodegradation. As shown in Fig. 1a, CAP was not significantly degraded (*p* > 0.05) by consortium CL at 1 h (stage 2). CAP was rapidly reduced with continuous production and accumulation of metabolites at 7 h and 13 h, which was the CAP-degrading middle stage (stage 3). At 21 h, the concentration of CAP was reduced to 11.9%, and the concentration of most metabolites began to decline from the peak level, which indicated the later stage (stage 4) of CAP biotransformation. There was no CAP remaining in the culture medium from 25 to 48 h, and most metabolites were completely degraded, which was defined as the terminal stage (stage 5). Finally, 77.0% of the soluble total organic carbon (TOC) introduced through CAP dosing was removed (Fig. 1a). Besides, along with the complete degradation of CAP by consortium CL, the antimicrobial activity of CAP was also thoroughly eliminated (Additional file 2: Fig. S1).

### Comprehensive characterization of CAP-degrading consortium via genome-centric metagenomics and cultivation-dependent approaches

To identify the main microbial players involved in CAP degradation, we assembled 24 metagenomic datasets of consortium CL. Eighteen metagenome-assembled genomes (MAGs) including 13 high-quality genomes (completeness > 94%, contamination < 3%) were recovered from over 377 GB of metagenomic data via metagenome binning (Additional file 1: Table S1). Fifteen of these MAGs were classified to the genus level, and three of them were annotated to the family level. The relative abundances of MAGs were calculated based on genome coverage, which was acquired by mapping the reads of metagenomic datasets to MAGs [20, 21]. MAG1 assigned to *Sphingomonas* was the most dominant MAG in consortium CL with a relative abundance of 64.3 ± 17.9%, implying that it was the most important core functional bacteria for CAP degradation in consortium CL (Fig. 1b). MAG2 (*Caballeronia* sp.), MAG3 (*Cupriavidus* sp.), and MAG5 (*Pigmentiphaga* sp.) were the three remaining dominant MAGs in consortium CL with average relative abundances of 10.6 ± 8.8%, 13.8 ± 7.7%, and 3.6% ± 0.6%, respectively (Fig. 1b). The relative abundance of all remaining MAGs was lower than 1.0%.

To gain deeper insights and conduct subsequent microbial interaction experiments based on predictions obtained via multi-omics, seven strains were isolated from consortium CL using mineral salt and R2A agar plates containing CAP. Their whole genomes were acquired through the combined application of Illumina sequencing and Oxford Nanopore sequencing (Additional file 1: Table S2, Additional file 2: Fig. S2a). Strains CL5.1, PC1, CLC6, RCL7, CLB6.2, PNB5, and CLB4 were classified as *Sphingomonas*, *Caballeronia*, *Cupriavidus*, *Chryseobacterium*, *Pigmentiphaga*, *Labrys*, and *Achromobacter* by genomic taxonomy classification using GTDB-Tk [22], respectively. A phylogenetic tree of isolated strain genomes and MAGs relative to 2170 GTDB reference genomes was constructed to determine their phylogenetic relationship. Strains CL5.1, PC1, CLC6, RCL7, CLB6.2, PNB5, and CLB4 clustered with MAG1, MAG2, MAG3, MAG4, MAG5, MAG6, and MAG7, respectively, which could indicate their extensive homology (Fig. 1c). The genomic homology of the isolated strains and MAGs was also confirmed by their high average amino acid identity (AAI) and average nucleotide identity (ANI) similarities (> 99.0%) (Additional file 2: Fig. S2b). The genomes obtained from pure culture isolation were superior to MAGs recovered from consortium CL in terms of completeness, the number of contigs, and N50 length (Additional file 2: Fig. S2c). Consequently, the genomes of the seven isolates replaced the corresponding MAGs as the reference genomes for the subsequent metatranscriptomic and proteomic analysis. Compared to the conventional pure culture isolation approach, metagenome assembly and binning could cover more strain genomes and even uncultured ones, which can provide full-spectrum genomic information for the complex microbial community [23]. For example, another 11 MAGs accounting for 2.46% of the total abundance of the CAP-degrading microbial community on average were recovered using the metagenomic approach, while no corresponding pure culture isolations could be obtained. These MAGs provided crucial clues in the elucidation of comprehensive metabolic pathways and biotransformation mechanisms of CAP as well as the functional interactions among the microorganisms in consortium CL. Therefore, pure culture isolation combined with second-generation and third-generation sequencing-based metagenomics is an effective approach to obtaining high-quality or even complete bacterial genomes [24], which are the key foundation for thoroughly deciphering CAP biotransformation mechanisms and microbial interactions.

### The transcriptional profile of CAP-degrading consortium during CAP metabolism

**Fig. 2:**
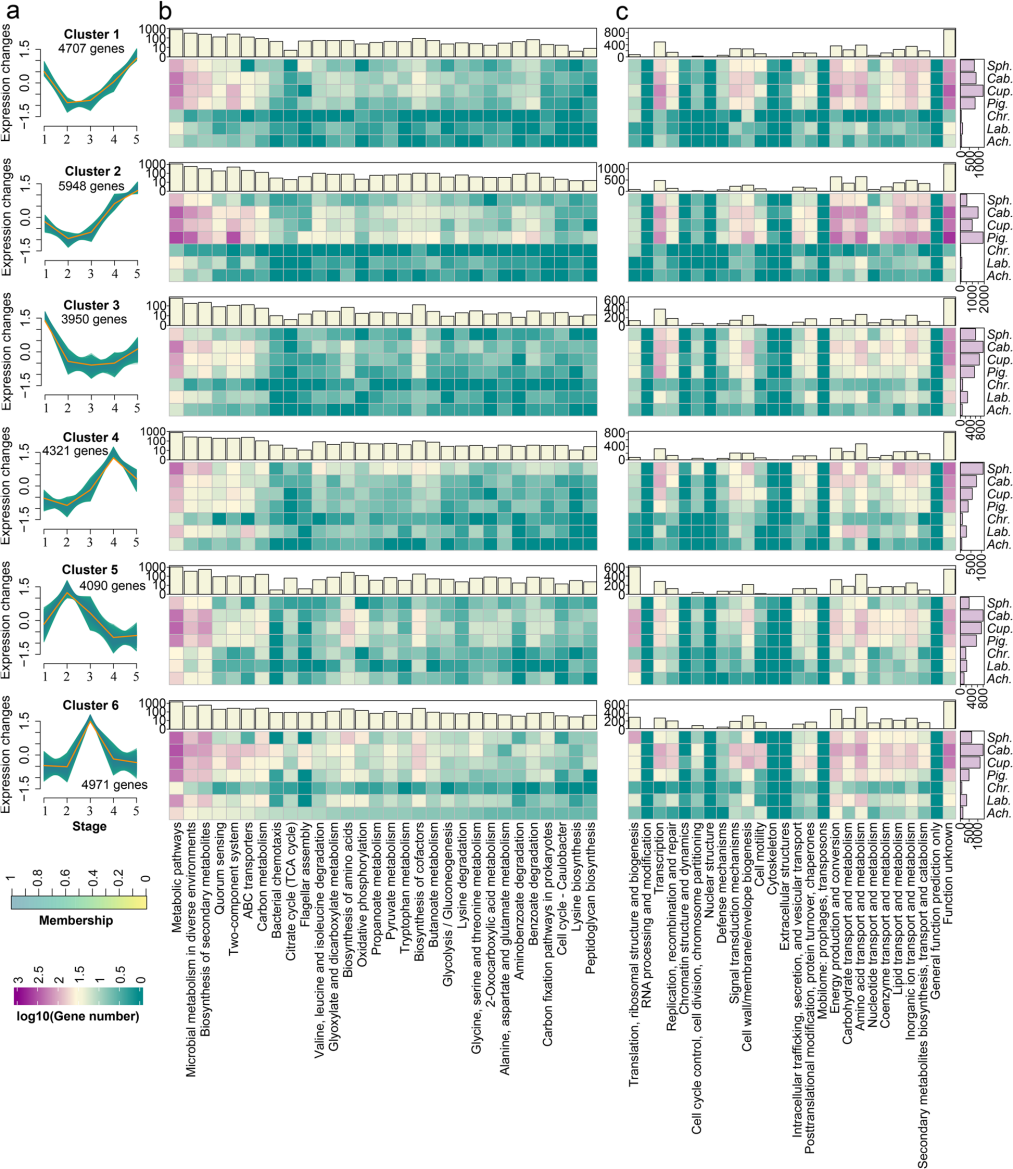
Gene expression patterns and functional annotation. **a** The expression patterns of temporal DEGs determined by time-series analysis. A high membership value indicates a high correlation of the gene expression with the cluster. **b** The KEGG annotation of temporal DEGs in each core species of consortium CL including *Sphingomonas* sp. (*Sph*.), *Caballeronia* sp. (*Cab*.), *Cupriavidus* sp. (*Cup*.), *Pigmentiphaga* sp. (*Pig*.), *Chryseobacterium* sp. (*Chr*.), *Labrys* sp. (*Lab*.), and *Achromobacter* sp. (*Ach*.). The top histogram indicated the total DEG number (log10 transformation) in each KEGG term in consortium CL. **c** The COG annotation of temporal DEGs in each core species of consortium CL. The top histogram indicates the total DEG number in each COG term, and the right histogram indicates the total DEG number in each strain

To elucidate the temporal transcriptional profile of consortium CL during CAP biotransformation, over 271 GB of time-series metatranscriptomic datasets were obtained from consortium CL. The strain-level transcriptional profiles of seven isolated key species (*Sphingomonas* sp., *Caballeronia* sp., *Cupriavidus* sp., *Pigmentiphaga* sp., *Chryseobacterium* sp., *Labrys* sp., and *Achromobacter* sp.) and 11 uncultured species (MAG8~MAG18) were acquired by assigning transcripts to their genomes according to the pipeline shown in Additional file 2: Fig. S3. In total, 27,987 temporal differentially expressed genes (DEGs) were identified via time-series analysis of the metatranscriptomic datasets based on a negative binomial noise model [25]. These temporal DEGs were assigned to six expression patterns via noise-robust soft clustering (Fig. 2a) [26]. The abundant temporal DEGs were involved in metabolism, biosynthesis, quorum sensing, two-component system, and ABC transporter-related pathways in genomes of seven key strains based on Kyoto Encyclopedia of Genes and Genomes (KEGG) annotation results (Fig. 2b). Clustering of orthologous groups (COGs) revealed that a large number of the temporal DEGs were involved in substrate transport and metabolism, energy production, and conversion during the biotransformation of CAP (Fig. 2c). The genes belonging to clusters 5 and 6 showed a reverse-“U”-shaped expression pattern similar to the variation pattern of the main metabolites (Fig. 1a). Most of these genes were upregulated in stages 2 or 3 of the CAP degradation process and had positive correlations with the dynamics of main CAP metabolites. This suggested that the genes belonging to clusters 5 and 6 might be involved in the biotransformation of CAP (Additional file 2: Fig. S4a). These genes were significantly enriched in KEGG pathways including carbon metabolism, energy metabolism, biosynthesis of amino acids, and translation (*p* < 0.05) (Additional file 2: Fig. S4b). CAP was the sole carbon and energy source for the consortium, and its presence promoted the metabolic activities of the entire community. The members of the consortium showed different physiological statuses and metabolic features at the transcriptional level, indicating their specific roles in CAP biotransformation. For instance, the presence of CAP exerted significant effects on cell motility-related pathways including flagellar assembly and bacterial chemotaxis in *Caballeronia* sp., *Cupriavidus* sp., and *Achromobacter* sp. during CAP rapid-degrading stage 3. Members belonging to the genera *Caballeronia*, *Cupriavidus*, and *Achromobacter* are highly motile via flagella [27, 28]. Thus, *Caballeronia* sp., *Cupriavidus* sp., and *Achromobacter* sp. were hypothesized to compete for nutrients via improving cell motility. Moreover, the temporal DEGs were bound up with the catabolism of CAP in the bacterial community and will be discussed in detail in “Comprehensive metabolic pathways and biotransformation mechanisms of CAP deciphered via the integrated multi-omics and cultivation-dependent approaches” and “The synergistic interactions on CAP mineralization among functional microorganisms.”

To sum up, the transcriptional profiling results revealed that CAP significantly triggered and promoted the metabolic activities of the key dominant members of consortium CL. This was the first time to reveal the temporal transcriptional expression profiles of the key members of a CAP-degrading consortium utilizing CAP as the sole carbon and energy source, which could provide important clues for the decryption of CAP biotransformation mechanisms as well as interactions among functional microorganisms.

### The roles of isolated strains in CAP biotransformation

**Fig. 3:**
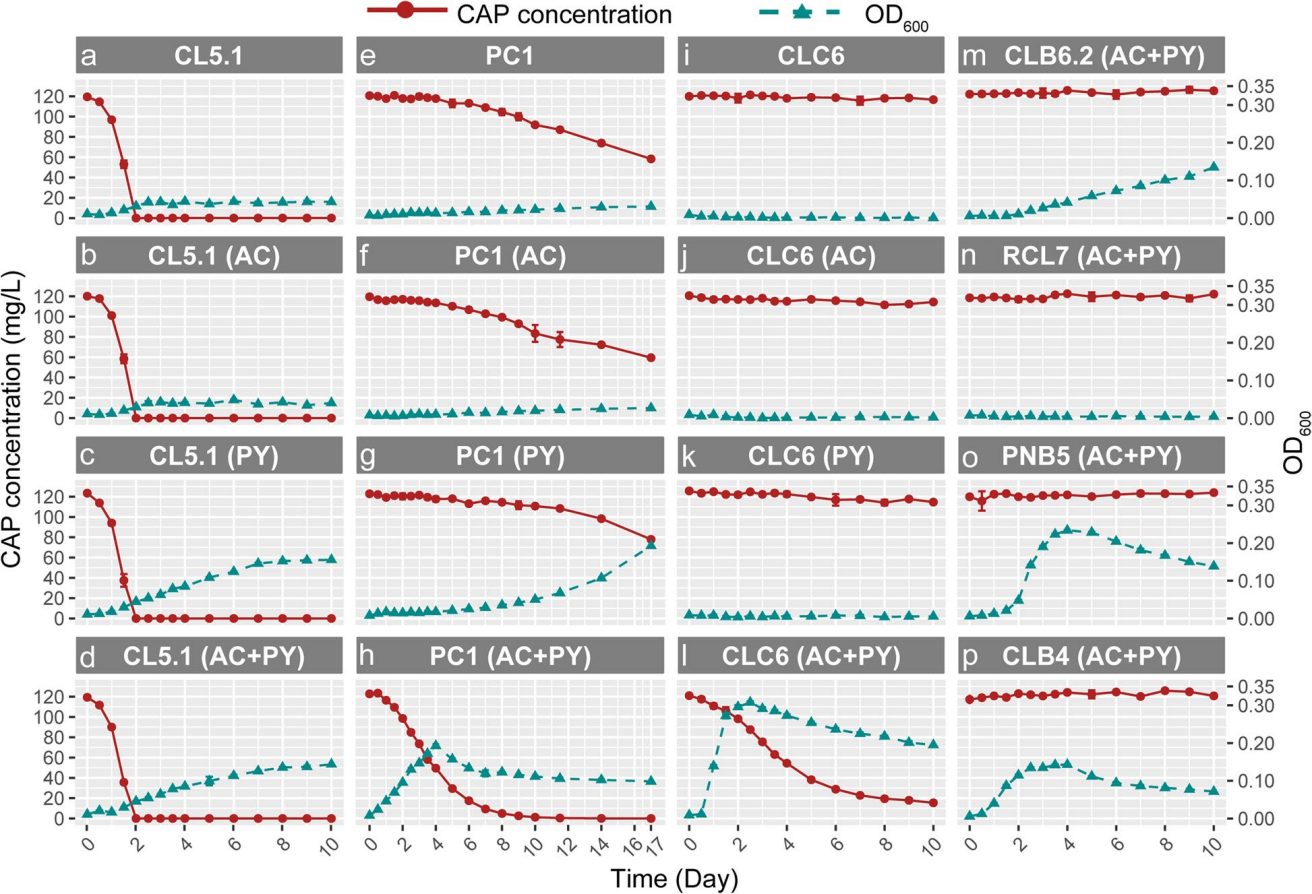
The biotransformation of CAP by isolated strains in axenic culture. The biotransformation of CAP by *Sphingomonas* sp. CL5.1 (**a**–**d**), *Caballeronia* sp. PC1 (**e**–**h**), and *Cupriavidus* sp. CLC6 (**i**–**l**) was tested under various nutrient conditions. AC and PY represented 30 mg/L ammonium chloride and 500 mg/L sodium pyruvate in the culture medium, respectively. The biotransformation of CAP by *Pigmentiphaga* sp. CLB6.2, *Chryseobacterium* sp. RCL7, *Labrys* sp. PNB5, and *Achromobacter* sp. CLB4 (**m**–**p**) was tested with the presence of 30 mg/L ammonium chloride and 500 mg/L sodium pyruvate in the culture medium. Data are presented as mean values ± SD (*n* = 3)

Based on the community-wide effects, the predicted varying roles of the key strains within the microbial consortium CL were investigated in microcosm experiments. CAP-degrading features of seven isolated strains were investigated under different nutritional conditions. *Sphingomonas* sp. CL5.1, *Caballeronia* sp. PC1, and *Cupriavidus* sp. CLC6 were identified as CAP degraders. *Sphingomonas* sp. CL5.1 was identified as the most vigorous degrader as it could completely degrade 120 mg/L CAP within 48 h even without needing an additional carbon or nitrogen source, which made it the core functional member for CAP biotransformation in the consortium CL (Fig. 3 a–d). *Caballeronia* sp. PC1 was another degrader that could utilize CAP as the sole carbon and nitrogen source but with a significantly lower degradation efficiency compared to *Sphingomonas* sp. CL5.1 (*p* < 0.001). After 17 days of cultivation, it could degrade 51.7% of CAP (Fig. 3e). Nutrient supplementation significantly promoted the degradation of CAP by *Caballeronia* sp. PC1 (*p* < 0.001) (Fig. 3 f–h). In the presence of both sodium pyruvate and ammonium chloride, *Caballeronia* sp. PC1 could degrade about 99.0% of CAP within 10 days (Fig. 3h). *Cupriavidus* sp. CLC6 was the final identified CAP-degrading bacterium although with a weak degradation ability. When CAP was supplied as the sole carbon or nitrogen source, the CAP degradation efficiency was less than 5.0%, and the biomass of *Cupriavidus* sp. CLC6 did not increase during 10-day cultivation (Fig. 3 i–k), indicating that it cannot utilize CAP as the sole carbon or nitrogen source to support its growth. However, when provided with both an additional carbon and nitrogen source, the strain could reproduce to a stationary phase in 2 days and degrade 87.3% of CAP within 10 days (Fig. 3l). *Pigmentiphaga* sp. CLB6.2, *Chryseobacterium* sp. RCL7, *Labrys* sp. PNB5, and *Achromobacter* sp. CLB4 were not able to degrade CAP even in the presence of additional carbon and nitrogen source (Fig. 3 m–p). Up to now, isolated CAP-degrading bacteria mainly included *Sphingobium* sp. CAP-1 [14], *Geobacter metallireducens* GS-15 [29], *Escherichia fergusonii* I-10-CHL [30], *Haemophilus influenzae* Rd KW20 [31], *Streptomyces* sp. 3022a [32], and *Clostridium acetobutylicum* [33]. Our findings expand the knowledge boundaries on CAP-degrading microbes at the class taxon level of Betaproteobacteria and could provide desirable strain resources for enhanced bioremediation of CAP-contaminated hotspot sites such as hospital wastewater, pharmaceutical wastewater, and livestock and poultry breeding wastewater. In addition, these isolated strains played crucial roles in verifying their functions regarding CAP biotransformation mechanisms and microbial interactions, which will be disclosed in the subsequent sections.

### Comprehensive metabolic pathways and biotransformation mechanisms of CAP deciphered via the integrated multi-omics and cultivation-dependent approaches

**Fig. 4:**
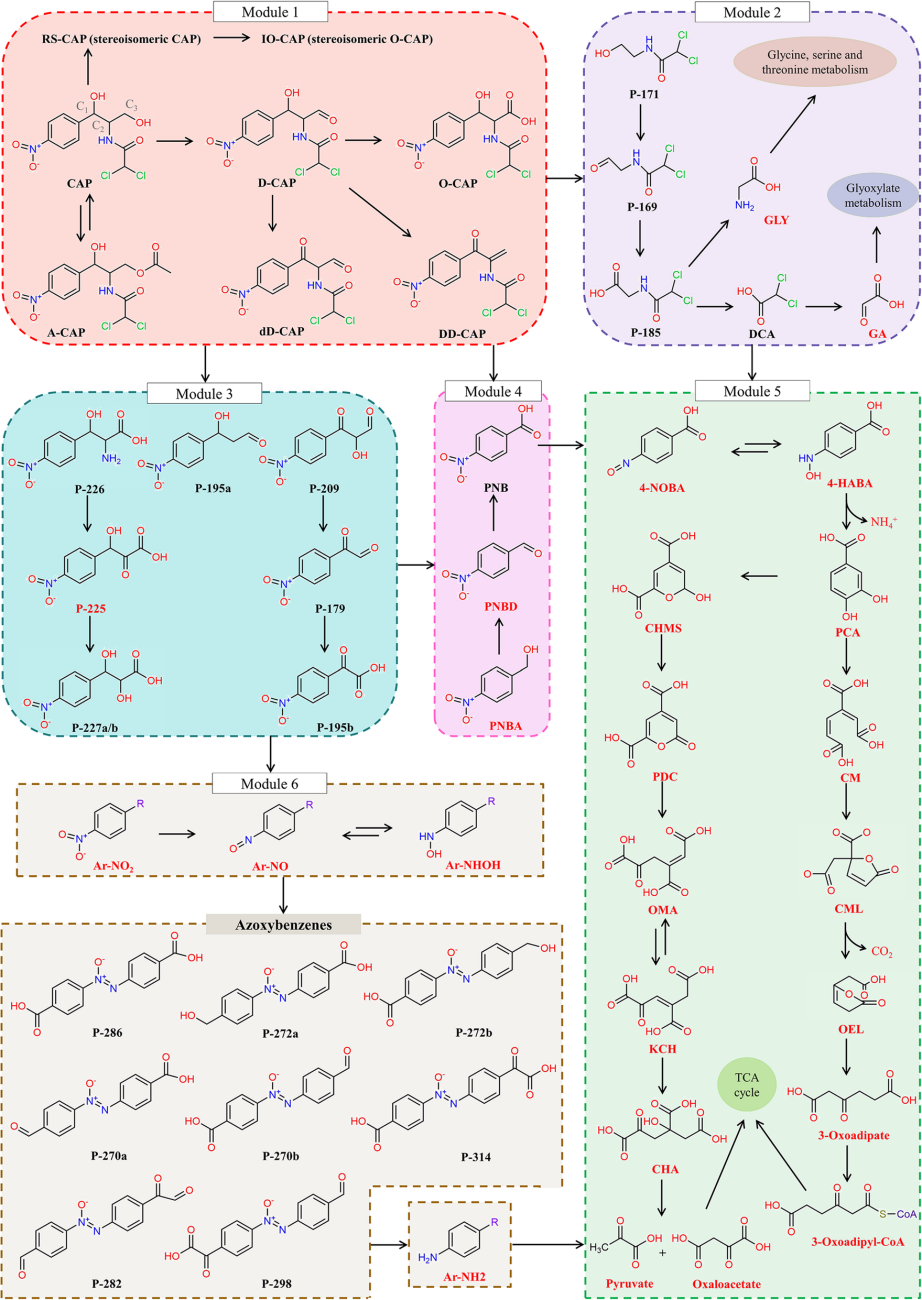
The proposed metabolic pathway of CAP. The metabolites in red color were deduced according to multi-omics rather than directly detected by HPLC-QTOF-MS

A comprehensive CAP metabolism pathway, related functional genes, and enzymes were thoroughly revealed by the combined application of proteomics, metagenomics, and metatranscriptomics, as well as cultivation-dependent approaches. As shown in Fig. 4, we defined six catabolic modules that jointly make up the entire CAP biotransformation pathway, which involves 27 metabolites directly detected by non-targeted high-performance liquid chromatography-quadrupole time-of-flight mass spectrometry (HPLC-QTOF-MS) analysis (Additional file 2: Fig. S5, Additional file 3) and 23 metabolites deduced by cultivation-independent multi-omics. The MS/MS spectra of 27 detected metabolites and 5 available metabolite standards, including information about *m/z* values of parent and fragment ions and retention time, were listed in Additional file 3. The detailed metabolic pathways and biotransformation mechanisms will be discussed as follows.

#### The oxidization at C_3_-OH of CAP

**Fig. 5:**
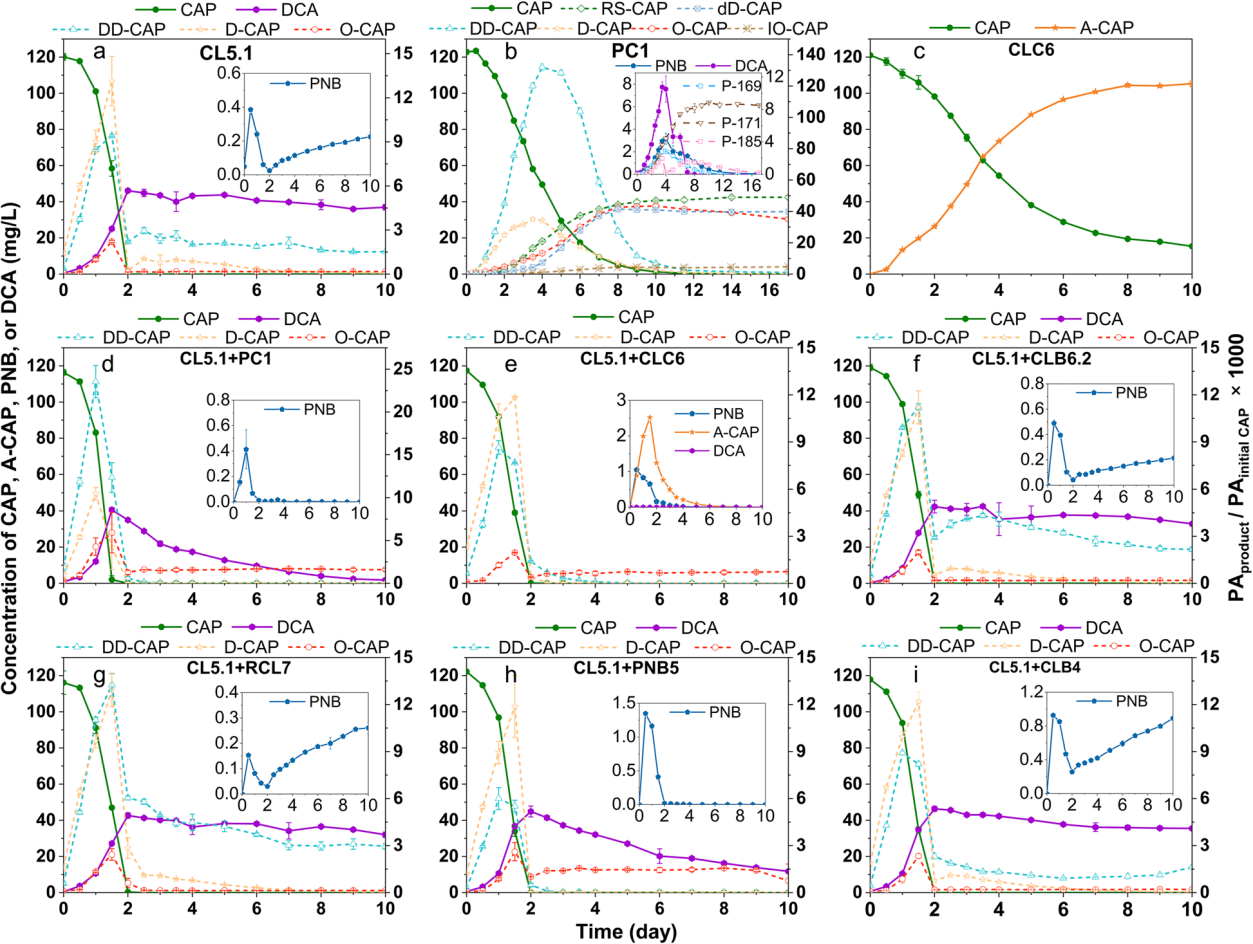
The dynamics of main CAP metabolites produced by isolated strains. **a**–**c** The dynamics of CAP metabolites produced by the axenic culture. *Sphingomonas* sp. CL5.1 was supplied with ammonium chloride as an additional nitrogen source. *Caballeronia* sp. PC1 and *Cupriavidus* sp. CLC6 were supplied with ammonium chloride and sodium pyruvate as additional nitrogen and carbon sources. **d–i** The dynamics of CAP metabolites produced by the co-culture of *Sphingomonas* sp. CL5.1 and other strains. The concentration of A-CAP, DCA, and PNB was absolutely quantified according to the chemical standards. Other metabolites were semi-quantified according to the proportion of their peak areas (PA) to the peak area of CAP at initial concentration. Data are presented as mean values ± SD (*n* = 3)

**Fig. 6:**
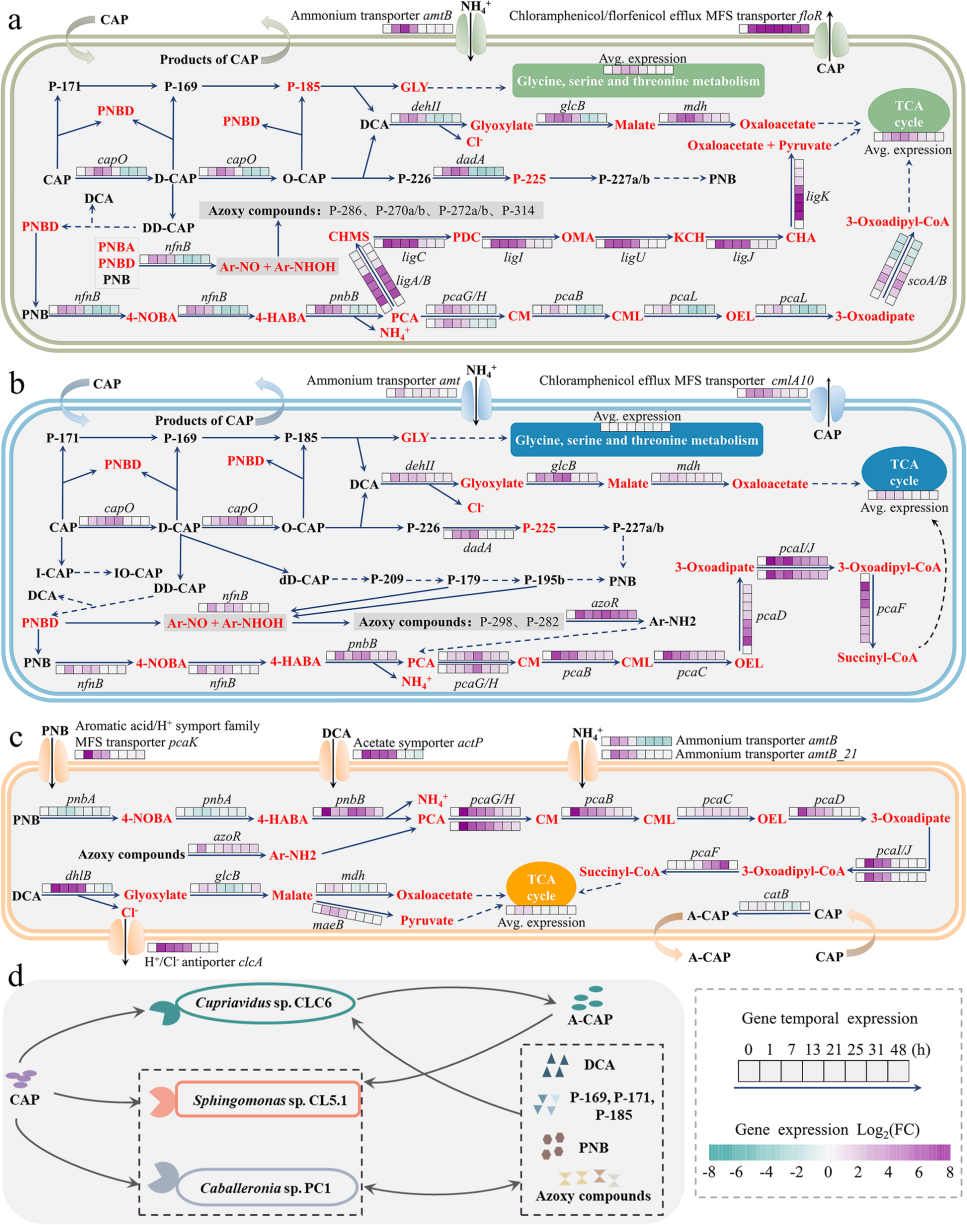
The proposed core genes involved in CAP biotransformation in cells and the interaction between members of the consortium in CAP metabolism. **a**–**c** Differential expression of genes involved in the CAP biodegradation in MAG1 (*Sphingomonas* sp.) (**a**), MAG2 (*Caballeronia* sp.) (**b**), and MAG3 (*Cupriavidus* sp.) (**c**). The little squares in various colors indicated the gene expression difference at different sampling times compared to that at 0 h (before CAP dosing). The gene expression was presented as Log_2_(FC) (*n* = 3). **d** Proposed substrate cross-feeding among key members in consortium CL

In catabolic module 1, C_3_-OH of CAP was oxidized to aldehyde producing D-CAP and then further oxidized to carboxyl producing O-CAP. D-CAP was also transformed to DD-CAP via dehydrogenation and dehydration. These biotransformation processes were discovered in *Sphingomonas* sp. CL5.1 and *Caballeronia* sp. PC1. In addition, the C_1_-OH of D-CAP could be dehydrogenized to dD-CAP by *Caballeronia* sp. PC1, which is a novel metabolic pathway first reported in this study. The accumulation of these products was observed during the stage of CAP rapid degradation (Fig. 5 a and b). However, the enzymes involved in these biotransformation processes were still unclear. According to the transcriptional information, a novel gene belonging to the glucose-methanol-choline (GMC) oxidoreductase superfamily was predicted to catalyze the two-step oxidations of CAP to O-CAP, and we designated this gene as *capO*. The GMC oxidoreductase superfamily harbors diverse enzymes with the function of catalyzing a wide variety of redox reactions such as the oxidation of an alcohol moiety to the corresponding aldehyde and the two-step oxidation of an alcohol moiety to the corresponding carboxylic acid [34]. For example, the choline dehydrogenase catalyzes the two-step oxidation of choline to glycine betaine [35]. In the current study, we found that *capO* presented a reverse-U expression pattern in *Sphingomonas* sp. (i.e., MAG1), and *Caballeronia* sp. (i.e., MAG2) during CAP biotransformation and especially the expression of *capO* in the main CAP degrader *Sphingomonas* sp. (i.e., MAG1) was significantly upregulated by 9.7-fold at 7 h compared to that at 0 h (*p* < 0.001) (Fig. 6 a and b). Moreover, the protein expression profile of *Sphingomonas* sp. CL5.1 in the presence of CAP was identified by proteomic sequencing. Consistent with the transcriptional pattern, the protein expression of *capO* in *Sphingomonas* sp. CL5.1 was upregulated by 2.74-fold (*p* < 0.001) at 34 h compared to that at 0 h (Additional file 1: Table S3). Therefore, the integrated multi-omics and cultivation-dependent approaches provided highly identical and solid clues for the candidate enzyme responsible for the oxidization at C_3_-OH of CAP. Moreover, the conversion from CAP to O-CAP was also found in *Sphingobium* sp. CAP-1, and it was consistent with our study that the expression of a GMC oxidoreductase protein homologous to capO was markedly upregulated by 3.20-fold in the presence of CAP [14]. To confirm the function of this candidate CAP oxidoreductase encoding gene *capO*, it was cloned and heterologously expressed in *Pseudomonas putida* KT2440. *Pseudomonas putida* KT2440 transformed with pBBR-*capO* could convert CAP into D-CAP and O-CAP with high efficiency, which was consistent with the above speculation (Additional file 2: Fig. S6). This finding expands our existing knowledge boundary on functional enzymes responsible for CAP metabolism in microbes. Besides, further investigation on the enzymatic characteristics, catalytic mechanism, and application potential should be conducted in the future.

#### The isomerization at C_2_ of CAP

Stereoisomerism was first discovered in the CAP biotransformation process in this study (Fig. 4 module 1). It is well-known that the C_1_ and C_2_ of chloramphenicol are chiral carbon atoms, and four stereoisomers exist, including 1R,2R-CAP (CAP, D-threo-chloramphenicol), 1S,2R-CAP (SR-CAP, D-erythro-chloramphenicol), 1R,2S-CAP (RS-CAP, L-erythro-chloramphenicol), and 1S,2S-CAP (SS-CAP, L-threo-chloramphenicol). RS-CAP was detected during CAP biotransformation initiated by *Caballeronia* sp. PC1 (Fig. 5b, Additional file 2: Fig. S7), presenting a novel CAP metabolic pathway through the isomerization at C_2_ of CAP. In addition, IO-CAP which is the oxidized product of RS-CAP at C_3_-OH was detected (Fig. 5b). It should be pointed out that IO-CAP is the stereoisomer of O-CAP. Among the four stereoisomers of chloramphenicol, only 1R,2R-CAP (CAP), which is the natural product from *Streptomyces venezuelae*, has significant antibacterial activity [36]. Thus, the discovered optical isomerization at C_2_ presents a novel bacterial resistance mechanism. This new finding remarkably deepened our understanding of the CAP metabolic processes as well as the CAP resistance mechanisms. The identification of the corresponding functional genes should be the focus of subsequent investigations.

#### The acetylation at C_3_-OH of CAP

Though *Cupriavidus* sp. CLC6 could not subsist on CAP alone, it could resist CAP by acetylating the C_3_-OH of CAP to produce chloramphenicol 3-acetate (A-CAP, module 1). In the presence of additional carbon and nitrogen sources, 105.3 mg/L A-CAP was detected in the culture medium, which exhibits *Cupriavidus* sp. CLC6’s strong CAP acetylation ability (Fig. 5c). The gene *catB* encoding CAP acetyltransferase in *Cupriavidus* sp. is responsible for CAP acetylation in consortium CL (Fig. 6c). Acetylated CAP is not able to bind to bacterial 50S ribosomal subunit, and acetylation is a common CAP bacterial resistance mechanism [37]. Thereby, CAP acetylation by *Cupriavidus* sp. was conducive to the decrease of toxic stress of CAP on the microbial community in the consortium [11].

#### The hydrolysis of the amide bond and the cleavage of C_1_ and C_2_

We hypothesize that there are two catabolic reaction types connecting module 1 to module 2 and module 3 (Fig. 4). These include the cleavage of C_1_/C_2_ and the hydrolysis of the amide bond. The formation of P-171, P-169, and P-185 by *Sphingomonas* sp. CL5.1 and *Caballeronia* sp. PC1 gave a valuable clue regarding the cleavage of C_1_ and C_2_ of CAP, D-CAP, dD-CAP, and DD-CAP. In addition, the hydroxyl of P-171 was probably further oxidized to produce P-169 and then P-185. It was observed that P-171, P-169, and P-185 were constantly generated and secreted to the culture medium within 4 days and were thereafter almost completely utilized by *Caballeronia* sp. PC1 (Fig. 5b). *Sphingomonas* sp. CL5.1 and *Caballeronia* sp. PC1 can produce 2,2-dichloroacetic acid (DCA) (Fig. 5 a, b), which was reported to be a common CAP transformation product [30, 38]. P-185 was inferred to be hydrolyzed into GLY and DCA by an uncertain hydrolase. The metatranscriptomic data revealed that the average expression of genes involved in the catabolism of glycine (GLY) in the glycine, serine, and threonine metabolism pathway was significantly upregulated in *Sphingomonas* sp. CL5.1 at 7 and 13 h (*p* < 0.001) (Fig. 6a). GLY can be converted to serine, CO_2_, and NH_4_^+^ via the glycine cleavage system coupled with hydroxymethyltransferase (glyA) (Additional file 2: Fig. S8). A series of enzymes in the glycine cleavage system respond to the high concentration of GLY. The upregulation of these enzymes in response to CAP dosing was observed at both RNA and protein levels in *Sphingomonas* sp. CL5.1 (Additional file 2: Fig. S8, Additional file 1: Table S3), which provided a valuable clue for GLY production during CAP biotransformation. DCA was predicted to be transformed to glyoxylate via dichlorination catalyzed by a 2-haloacid dehalogenase (dehII or dhlB) [39], followed by being metabolized to malate and oxaloacetate via successive catalysis of malate synthase (glcB) and malate dehydrogenase (mdh) [40] (Fig. 6 a, b, c). Finally, it was converted into energy in the tricarboxylic acid cycle (TCA) in *Sphingomonas* sp., *Caballeronia* sp., and *Cupriavidus* sp. (Fig. 6 a, b, c).

The production of P-226 indicated the hydrolysis of the amide bond of metabolites in Module 1 (Fig. 4, Additional file 2: Fig. S9). For instance, the amide bond of O-CAP was most likely hydrolyzed to P-226 and DCA in *Sphingomonas* sp. CL5.1 and *Caballeronia* sp. PC1 (Additional file 2: Fig. S9 a and b). Previous studies proved that the amide bond of CAP was prone to be broken down by microbial esterases [12]. P-226 was inferred to be further deaminized to P-225 via oxidative catalysis of a D-amino acid dehydrogenase (dadA) (Fig. 6 a, b). DadA can catalyze the oxidative deamination of D-amino acids or their analogs into corresponding oxoacids with broad substrate specificity [41]. We hypothesize that P-225 was probably reduced to P-277a and P-277b by an uncertain ketol reductase in *Sphingomonas* sp. CL5.1 and *Caballeronia* sp. PC1. P-277a and P-277b were stereoisomers with the same mass and identical fragment ion spectra, but with different retention times. In common, ketone reduction can convert prochiral ketones into chiral alcohols [42]. Thus, the prochiral ketone of P-225 was ready to be reduced to chiral products. Besides, P-209, P179, P-195a, and P-195b were detected to be produced by *Caballeronia* sp. PC1 (Additional file 2: Fig. S9b). We hypothesize that P-209 was converted from dD-CAP via oxidative deamination and ketol reduction and was thereafter converted to P-179 and P-195b via several reactions (Fig. 4 module 3, Fig. 6b).

#### The cleavage of PNB

The nitrophenyl of CAP and its analog products in module 1 and module 2 were finally converted to 4-nitrobenzoic acid (PNB) via several reactions. For instance, CAP, D-CAP, dD-CAP, and DD-CAP could be cleaved into 4-nitrobenzaldehyde (PNBD) via the oxidative cleavage of C_1_ and C_2_ and could finally be oxidized to 4-nitrobenzoic acid (PNB) (Module 4). Several studies have shown that PNB was one of the main intermediate metabolites from CAP conversion [14, 30, 32]. Though no intermediate products of PNB were detected in this study, a metabolic pathway was established according to the metagenomic and metatranscriptomic information (module 5 of Figs. 4 and 6 a–c). The nitro group of PNB was reduced to an amino group and then to a hydroxylamine group producing 4-nitrosobenzoate (4-NOBA) and 4-hydroxylaminobenzoate (4-HABA) via catalysis through a nitroreductase (nfnB or pnbA) [43, 44]. Then, the product 4-HABA was transformed to protocatechuate (PCA) catalyzed by 4-hydroxylaminobenzoate lyase (pnbB) [45]. In general, PNB can be mineralized via PCA cleavage pathways in aerobic microbes [46, 47]. It is well-known that PCA is a pivotal intermediate in the downstream metabolic route for the degradation of a variety of aromatics, and its ring can be cleaved in three modes including 4,5-cleavage (meta-cleavage), 3,4-cleavage (ortho-cleavage), and 2,3-cleavage [48]. In the present study, two types of gene clusters participated in the ortho-cleavage and meta-cleavage pathways of PCA in consortium CL (Additional file 2: Fig. S10).

*Sphingomonas* sp. CL5.1, *Caballeronia* sp. PC1, and *Cupriavidus* sp. CLC6 had the ability of PNB utilization (Additional file 2: Fig. S11a-c). They carry the *nfnB*/*pnbA* and *pnbB* genes involved in the reductive conversion of PNB to PCA (Fig. 6 a–c). Based on the annotated gene clusters relating to the PCA metabolism (Additional file 2: Fig. S10), *Sphingomonas* sp. CL5.1 could mineralize PCA via both ortho- and meta-cleavage pathways, while *Caballeronia* sp. PC1 and *Cupriavidus* sp. CLC6 could only mineralize PCA via the ortho-cleavage pathway (Fig. 6 a, b, c). The genes involved in the metabolism of PCA in *Sphingomonas* sp., *Caballeronia* sp., and *Cupriavidus* sp. were expressed in a reverse-U pattern, implying that they indeed mineralized PCA in the corresponding pathway. Moreover, the proteins responsible for PNB metabolism were also significantly upregulated in the presence of CAP according to results of the comparative proteomic analysis in *Sphingomonas* sp. CL5.1, which further confirmed the proposed metabolic processes of PNB (Additional file 1: Table S3). Besides, *62-47* sp. (MAG15) was predicted to be capable of PNB biotransformation, as it carried *nfnB*, *pnbB*, and genes involved in ortho-cleavage of PCA. *Bosea* sp. (MAG8) was predicted to conduct ortho-cleavage of PCA (Additional file 2: Fig. S10). *Pigmentiphaga* sp. was predicted to be capable of both ortho-cleavage and meta-cleavage of PCA. Nevertheless, the lack of genes involved in PNB nitroreduction was a disadvantage for *Bosea* sp. and *Pigmentiphaga* sp. in carbon utilization of PNB, which probably resulted in their low relative abundance in the consortium CL.

#### The formation of azoxybenzenes

Surprisingly, eight biotransformation products including P-314, P-298, P-286, P-282, P-272a, P-272b, P-270a, and P-270b produced by *Sphingomonas* sp. CL5.1 and *Caballeronia* sp. PC1 were inferred to be azoxybenzenes which are dipolar N-oxides of azobenzene compounds (Fig. 4 module 6). Besides, P-286 was identified to be 4,4′-azoxydibenzoic acid as it owned the same retention time, molecular weight, and MS/MS spectrum features determined by HPLC-QTOF-MS (Additional file 3). A variety of microbes and plants can produce azoxybenzenes, and they may function as deterrents, virulence factors, or means to defend the habitat [49]. For instance, it has been determined that *Entomophthora virulenta* could produce 4,4′-azoxydibenzoic acid as a secondary metabolite [50]. Boer et al. found that P-286 was a metabolite of CAP degradation in eye drops [51]. Nitrobenzene compounds are ingredients for the biosynthesis of azoxybenzenes in microorganisms [49]. As shown in Fig. 4 (module 3, module 4, and module 6), nitrobenzene products (Ar-NO_2_) including P-179, P-195b, and PNB supposedly formed into nitrosobenzene compounds (Ar-NO) first and then into hydroxylaminobenzene compounds (Ar-NHOH) under the catalysis of the nitroreductase (nfnB) based on the nitroaromatic compound degradation pathway reported previously [47]. Gene *nfnB* located in the genome of *Sphingomonas* sp. and *Caballeronia* sp. was observably upregulated at stage 3 of CAP degradation (*p* < 0.001). Nitrosobenzene compounds and hydroxylaminobenzene compounds have been reported to be an important intermediate to dimerize to azoxy bond azoxybenzenes in bacteria, and the azoxy bond formation was a nonenzymatic process [52]. The azoxy bond is a crucial chemical bond for the synthesis of pharmaceuticals, dyes, etc. [49, 52]. This finding implies the potential of biosynthesis of the azoxy bond on the base of nitrobenzene compounds, which probably opens a new route for azoxybenzenes biosynthesis.

In summary, the initial biotransformation steps involved in this comprehensive microbial metabolism pathway were the oxidization at the C_1_-OH and C_3_-OH groups, the isomerization at C_2_, and the acetylation at C_3_-OH of CAP. Among them, the isomerization at C_2_ is an entirely new biotransformation pathway of CAP discovered in this study. Notably, the structures of these products (e.g., IO-CAP, D-CAP, dD-CAP, and DD-CAP) first reported in this study without available standards still require further verification through other multiple analysis methods such as nuclear magnetic resonance. We also confirmed that a novel glucose-methanol-choline oxidoreductase *capO* was responsible for the oxidization of the C_3_-OH group of CAP in *Sphingomonas* sp. CL5.1 and *Caballeronia* sp. PC1. Besides, the subsequent biotransformation steps, corresponding catalyzing enzymes, and the microbial players responsible for each step were comprehensively deciphered via integrated multi-omic analysis and cultivation-dependent approaches.

### The synergistic interactions on CAP mineralization among functional microorganisms

The synergistic interactions of the isolated strains were investigated via co-culture experiments. When CAP was supplied as the sole carbon source, the co-cultures did not perform better than axenic cultures regarding CAP biodegradation efficiency (Fig. 3 and Additional file 2: Fig. S12). Nevertheless, synergism of the co-cultures *Sphingomonas* sp. CL5.1 with *Caballeronia* sp. PC1 or *Cupriavidus* sp. CLC6 was significantly reflected by the mineralization aspect (*p* < 0.05). The mineralization rate of CAP in the axenic culture of *Sphingomonas* sp. CL5.1 was about 52.2% after 10-day cultivation (Additional file 2: Fig. S13a). The co-culture of *Sphingomonas* sp. CL5.1 and *Caballeronia* sp. PC1 removed 76.4% of soluble TOC in 2 days, which was significantly higher than that in axenic culture (*p* < 0.05) (Additional file 2: Fig. S13f). The co-culture of *Sphingomonas* sp. CL5.1 and *Cupriavidus* sp. CLC6 removed 73.6% of soluble TOC after 3-day cultivation which was also markedly higher than the axenic culture (*p* < 0.05) (Additional file 2: Fig. S13g). There was still about 37.0 mg/L DCA remaining in the axenic culture of strain CL5.1, while there was no detectable DCA remaining in the co-cultures of strain CL5.1 and strains CLC6/PC1 (Fig. 5 a, d, e). *Caballeronia* sp. PC1 and *Cupriavidus* sp. CLC6 were talented degraders of DCA. Especially, *Cupriavidus* sp. CLC6 could completely degrade 50 mg/L DCA within 24 h (Additional file 2: Fig. S11 b, c). These two strains assisted *Sphingomonas* sp. CL5.1 to degrade DCA converted from CAP and thus made great contributions to mineralization of CAP in co-cultures or consortia.

According to the above metabolic, genomic, proteomic, metagenomic, and metatranscriptomic information, we proposed an interaction network on feeding among key members of consortium CL. *Sphingomonas* sp., *Caballeronia* sp., and *Cupriavidus* sp. were the keystone species and the primary degraders of CAP in the consortium CL. They converted CAP into RS-CAP, O-CAP, dD-CAP, DD-CAP, and A-CAP via isomerization, oxidization, and acetylation and thus detoxified CAP for other members of the community (Fig. 6 a–c). These biotransformation products were further cleaved into the downstream metabolites including PNB, DCA, azoxybenzenes, and dichloroacetyl compounds (P-171, P169, and P-185) by *Sphingomonas* sp. CL5.1 and *Caballeronia* sp. PC1 (Fig. 6d). A part of these downstream metabolites was utilized by the producers, while the other part of them was released to the culture medium to be competitively assimilated by other members of the community. *Cupriavidus* sp. CLC6 mainly fed on DCA, while it could not catabolize A-CAP produced by itself (Fig. 6d). The expression of acetate symporter (*actP*) located at the genome of *Cupriavidus* sp. was significantly upregulated (*p* < 0.05) during CAP biotransformation. Gene *actP* is responsible for the transmembrane transport of acetate [53]. Thus, upregulation of this gene implied the assimilation of DCA by *Cupriavidus* sp., which promoted its predominant role in the community. As the core CAP degraders, *Sphingomonas* sp., *Caballeronia* sp., and *Cupriavidus* sp. could reproduce rapidly due to their advantage in nutrient utilization and thus be dominant in the microbial community.

Collectively, the synergistic collaboration of members in the consortium CL contributed to the biotransformation and mineralization of CAP. Meanwhile, they competed for substrates for growth and reproduction resulting in differences in their population sizes. The interactions between bacteria are complicated processes, and only limited knowledge regarding them can be acquired based on cultivation-based techniques, especially since only a few bacteria can be isolated [17, 54]. We demonstrated that the integrated application of multi-omics and cultivation-based approaches is effective to elucidate interspecies interaction-based environmental bioprocesses.

## Conclusions

In summary, different from the biotransformation studies using pure cultures, investigating the chloramphenicol biotransformation by microbial consortia could truly reflect the metabolic mechanisms without bias in the engineered or natural environments, where biotransformation processes are especially dependent on synergistic interactions and metabolite exchanges among microbes. Herein, this study successfully provides the first demonstration of obtaining a comprehensive view of the biotransformation mechanisms and the bacterial interactions in a bacterial community feeding on CAP via an integrated multi-omics approach and cultivation-based techniques, which afforded desirable strain and enzyme resources and a theoretical foundation for developing enhanced bioremediation of CAP-contaminated hotspot sites such as hospital wastewater, pharmaceutical wastewater, and livestock and poultry breeding wastewater.

## Methods

### Consortium enrichment and strain isolation

Consortium CL subsisting on CAP was originally enriched from the activated sludge of a local wastewater treatment plant, which had been described in detail in the previous study [19]. Consortium CL was cultured and passaged in a mineral salt culture medium (MSM) with 120 mg/L CAP and 30 mg/L NH_4_Cl for about 1.5 years. The compositions of MSM were as follows: KH_2_PO_4_, 7.0 g/L; Na_2_HPO_4_, 0.67 g/L; CaCl_2_, 0.015 g/L; and MgSO_4_, 0.097 g/L; metal trace elements are as follows: MnSO_4_•H_2_O, 1 mg/L; FeSO_4_•7H_2_O, 1 mg/L; Na_2_MoO_4_•2H_2_O, 0.25 mg/L; and CuCl_2_, 0.25 mg/L. The passage was conducted once a week. Cultivation was conducted under 120 rpm shaking incubation at 25 °C in triplicate. The culture of consortium CL was sampled for nucleic acid extraction and chemical analysis at 0 (before CAP dosing), 1, 7, 13, 21, 25, 31, and 48 h after CAP dosing, which covered all stages of CAP biodegradation. The samples for nucleic acid extraction were stored at −80 °C after flash freezing with liquid nitrogen.

Three types of media, each supplemented with 120 mg/L CAP, were used for strain isolation via the spread plate method:

- Medium A was MSM with 30 mg/L NH_4_Cl.
- Medium B was MSM with 30 mg/L NH_4_Cl and 500 mg/L sodium pyruvate.
- Medium C was R2A.

### CAP biodegradation and co-metabolism experiments of CAP, PNB, and DCA

CAP degradation properties of individual cultures and co-cultures were tested. Strain RCL7 was cultured in R2A medium with 120 mg/L CAP, and the other isolated strains were cultured in MSM with 120 mg/L CAP, 500 mg/L sodium pyruvate, and 30 mg/L NH_4_Cl. After 3-day cultivation, the cells were collected by centrifugation at 10,000 g for 5 min, and the cell precipitate was washed by MSM two times. Then, the precipitate was resuspended in MSM and inoculated to 150 mL MSM at the initial optical density at 600 nm (OD_600_) of 0.03. As for co-cultures, two strains were inoculated at initial equivalent biomass, and the total OD_600_ was also 0.03. The initial CAP concentration was 120 mg/L. A group without bacteria inoculation was set as the control. All batch experiments were conducted in triplicate. The culture liquid was sampled for chemical analysis during the experiment. The biomass of strains was quantified by a microplate reader Infinite M200 (Tecan, Switzerland). The soluble total organic carbon (TOC) was measured by a TOC-L analyzer (Shimadzu, Japan). The co-metabolism of CAP, PNB, and DCA by isolated strains was investigated by a similar process to CAP biodegradation experiments. The initial concentrations of CAP, PNB, and DCA were all 50 mg/L, and 30 mg/L NH_4_Cl was supplied as the additional nitrogen source.

### Antimicrobial activity test

The antimicrobial activity of CAP and its product residues in the culture medium was determined by an antimicrobial activity test using a CAP susceptible strain *Staphylococcus aureus* ATCC 25923. After inoculation with consortium CL, the MSM solution containing CAP with an initial concentration of 120 mg/L was collected and sterilized by filtration with a 0.22 μm sterile syringe filter (Millipore, USA). The filtrated samples mixed with equal volume Mueller-Hinton (MH) broth were used as the culture medium for the antimicrobial activity test. The mixture of MH broth and MSM without CAP was included as the control sample. The antimicrobial activity test was conducted in 96-well plates. *Staphylococcus aureus* ATCC 25923 after overnight incubation was inoculated into the mixed culture medium in 96-well plates. After cultivation at 37 °C for 20 h, the biomass of *Staphylococcus aureus* ATCC 25923 in 96-well plates was determined by a microplate reader Infinite M200 (Tecan, Switzerland).

### Metabolite analysis using HPLC-QTOF-MS

CAP and its metabolites were scanned and quantified by HPLC-QTOF-MS (Impact II™, Bruker, Germany) in both negative (ESI−) and positive (ESI+) ionization modes. Actually, CAP and its products were quantified in ESI− as all of them were more sensitive in ESI− mode. A Thermo Hypersil GOLD column (100 mm × 2.1 mm, 1.9 μm) was used for chromatographic separation in HPLC. The parameters of HPLC and mass spectrometer were set as the previous description except for the HPLC flow rate which was modified to 0.2 mL/min [13]. Metabolites were determined by comparison of MS chromatographs of the control group and the experimental group samples in DataAnalysis (v4.4). The structures of metabolites were determined through product ion scans under collision energy of 10~40 eV. The sodium formate solution at 1 mM was used for QTOF calibration. D-threo-chloramphenicol (Sigma-Aldrich, USA), D-erythro-chloramphenicol (J&K Scientific, China), L-erythro-chloramphenicol (J&K Scientific, China), L-threo-chloramphenicol (J&K Scientific, China), Chloramphenicol 3-acetate (TRC, Canada), 4-nitrobenzoic acid (Sigma-Aldrich, USA), 2,2-dichloroacetic acid (J&K Scientific, China), and 4,4′-azoxydibenzoic acid (Macklin, China) were used as standards for identification and quantification of CAP and its products. CAP and its metabolites were quantified using QuantAnalysis (v4.4) through the external standard method.

Chiral HPLC-MS analysis of four CAP stereoisomers was performed using HPLC-QTOF-MS with a CHIRALPAK AGP column (150 mm × 3 mm, 5 μm). The main parameters of HPLC were set as follows: column temperature, 21 °C; flow rate, 0.3 mL/min; and injection volume, 5 μL. Eluent A was Milli-Q water containing 5 mM ammonium acetate, and Eluent B was methanol. The elution gradient profile was as follows:

**Table:** 

Time	Eluent A	Eluent B
0 min	97%	3%
8 min	97%	3%
8.1 min	90%	10%
16 min	90%	10%
16.1min	97%	3%
22 min	97%	3%

### Total DNA extraction and genomic sequencing of isolated strains

The cells of isolated strains at the exponential growth stage were collected for total DNA extraction. Total DNA was extracted using DNeasy UltraClean Microbial Kit (QIAGEN, USA) according to the manufacturer’s instructions. DNA for Oxford Nanopore sequencing was purified by 1% agarose gel electrophoresis and Monarch DNA Gel Extraction Kit (NEB, USA).

Oxford Nanopore sequencing and Illumina sequencing were combined to obtain the complete genomes of isolated strains. Oxford Nanopore sequencing library was constructed using the Ligation Sequencing 1D kit (SQK-LSK109) (Oxford Nanopore, USA) according to the manufacturer’s protocol. Qubit® 2.0 Fluorometer (Life Technologies, USA) was used to quantify the constructed library. The library was sequenced using a PromethION (Oxford Nanopore Technologies, UK) with the R9.4 flow cell at NextOmics (Wuhan, China), and the sequencing depth was about 1 Gb.

The genomic Illumina library was constructed using the NEBNext® Ultra™ DNA Library Prep Kit for Illumina (NEB, USA) according to the manufacturer’s instructions. The library was sequenced on the Illumina NovaSeq platform (paired-end 150 bp) with a sequencing depth of about 2 Gb at NextOmics (Wuhan, China).

### Total DNA extraction and metagenomic sequencing of consortium CL

Total DNA extraction of consortium CL samples was performed using FastDNA™ Spin Kit for Soil (MP Biomedicals, USA) according to the manufacturer’s protocol. The extracted DNA was qualified by 1% agarose gel electrophoresis and quantified by Qubit® 2.0 Fluorometer (Life Technologies, CA, USA).

The metagenomic library was constructed using NEBNext® Ultra™ DNA Library Prep Kit for Illumina (NEB, USA) according to the manufacturer’s instructions and then was sequenced on the Illumina NovaSeq platform (paired-end 150 bp) with a sequencing depth of about 10 Gb at Novogene (Tianjin, China).

### Total RNA extraction and meta-RNA sequencing of consortium CL

Total RNA of consortium CL was extracted using RNeasy Mini Kit (QIAGEN, USA), and the remaining genomic DNA was digested with RNase-Free DNase Set (QIAGEN, USA). To improve bacterial cell lysis efficiency, the samples were processed as the following steps before extraction according to the manufacturer’s instructions: 200 μl of 15 mg/mL lysozyme was added to the samples, and then, the samples were incubated on ice for 10 min. After incubation, 700 μl lysis buffer containing 1% (v/v) β-mercaptoethanol and an appropriate amount of 0.1 mm glass beads (BioSpec Products, USA) was added to the samples, and then, the lysate was homogenized with a homogenizer (JXFSTPRP-64, Shanghaijingxin Experimental Technology, China). The extracted RNA was stored at −80 °C before sequencing. The ribosomal RNA was removed from RNA samples, and a strand-specific library was constructed using NEBNext® Ultra™ Directional RNA Library Prep Kit for Illumina (NEB, USA). Metatranscriptomic sequencing was performed on the Illumina NovaSeq platform (paired-end 150 bp) with a sequencing depth of about 10 Gb at Novogene (Tianjin, China).

### Sample preparation, protein extraction, and proteomic sequencing of strain CL5.1

*Sphingomonas* sp. CL5.1 was inoculated into MSM containing 1 g/L sodium pyruvate and 30 mg/L NH_4_Cl. After 3-day cultivation, strain CL5.1 was inoculated into 400 mL fresh MSM containing 1 g/L sodium pyruvate and 30 mg/L NH_4_Cl as the control group and MSM containing 120 mg/L CAP and 30 mg/L NH_4_Cl as the CAP-treatment group. This batch test was conducted in duplicate. The cells of strain CL5.1 in the CAP-treatment group were collected at 34 h (CT_34_) and 72 h (CT_72_) when about 66.6% and 99.8% of CAP were degraded, respectively. The cells in the group without CAP were collected as the control samples at 34 h. These collected samples were stored at −80 °C after flash freezing with liquid nitrogen. The protein extraction and tandem mass tag (TMT) labeling proteomic sequencing were performed as the method described in the previous study [38].

### Genome assembly of isolated strains

The original FAST5 files generated by Oxford Nanopore sequencing were converted into fastq files using Guppy (v3.4.1). Then, the data were filtered by Filtlong (https://github.com/rrwick/Filtlong) with a minimum average quality threshold of 10 and a minimum length threshold of 500 bp. The assemblies of the filtered data were obtained using Flye (v2.6) [55]. The raw data generated from Illumina sequencing were filtered by fastp (v0.20.1) [56]. The draft assemblies were improved by Medaka (v1.2.3) (https://github.com/nanoporetech/medaka), Rcon (v1.4.3) (https://github.com/isovic/racon), and Pilon (v1.23) [57] reference to the Illumina short reads to obtain high-quality Nanopore long read assemblies (NAGs) of strains.

### Metagenome assembly and MAGs recovery in consortium CL

The integration analysis process of multi-omics data was shown in Additional file 2: Fig. S3. First, the raw metagenomic data were filtered by fastp (v0.20.1) [56] and then were assembled using metaSPAdes (v3.13.2) [58]. MAGs were recovered from the assemblies using BASALT (https://github.com/EMBL-PKU/BASALT) which integrates Maxbin2 (v2.2.7) [59], MetaBAT2 (v2.12.1) [60], and CONCOCT (v1.1.0) [61]. The contamination contigs were removed from MAGs by RefineM (v0.0.22) based on genomic properties and taxonomic assignments to improve their completeness [62]. The completeness and contamination of MAGs were assessed by CheckM (v1.1.2) [63]. MAGs de-replication was performed using drep (v2.0.0) [64]. The filtered reads of each sample were mapped against MAGs using Bowtie2 (v2.3.5.1)  [65] to obtain genome coverage, and then the relative abundance of each MAG was calculated based on the output file of read mapping using the script calculate_breadth.py provided by Olm et al. [20, 21].

### Taxonomy classification, phylogenetic analysis, and functional annotation of MAGs and NAGs

The taxonomies of all genomes including NAGs and MAGs were classified by GTDB-Tk (v1.0.2) according to 120 single-copy marker proteins [22]. A phylogenetic tree of NAGs, MAGs, and related 2170 reference genomes of GTDB based on 120 single-copy marker proteins for bacteria was constructed using the maximum likelihood method via FastTree (v2.1.11) [66]. The phylogenetic tree was visualized by iTOL (v5) [67]. The AAI and ANI similarities between NAGs and MAGs were calculated by CompareM (v0.0.23, https://github.com/dparks1134/CompareM) and pyani (v0.2.10, https://github.com/widdowquinn/pyani).

The coding DNA sequences (CDSs) of NAGs and MAGs were predicted by prodigal (v2.6.3) [68]. tRNA and rRNA were predicted by Prokka (v1.14.5) [69]. The protein function of CDSs was annotated by a blast search against the UniProtKB database. Clusters of orthologous group (COG) terms of CDSs were annotated by eggNOG-mapper (v2) [70]. The Kyoto Encyclopedia of Genes and Genomes (KEGG) annotation of protein sequences was performed by KofamKOALA [71].

### Time-series metatranscriptomic analysis

As NAGs had a significant quality advantage over MAGs, so NAGs substituted the corresponding MAGs as reference genomes for metatranscriptomic analysis. The raw metatranscriptomic data were filtered by fastp (v0.20.1) [56]. The filtered reads were mapped to the reference genomes by Bowtie2 (v2.3.5.1) [65], and mapped reads were counted using HTseq (v0.13.5) [72] to obtain the read count of each gene. The temporal differentially expressed genes (DEGs) were identified by time-series analysis of read counts using ImpulseDE2 with an adjusted *p*-value ≤ 0.05 [25]. The read counts were normalized to transcripts per million (TPM). The temporal DEGs were clustered to different expression patterns with the TPM table via Mfuzz [26]. The fold change (FC) and differences in gene expression between two samples were calculated by DEseq2 [73].

### Comparative proteomic analysis in Sphingomonas sp. CL5.1

The proteins predicted from the completed genome of strain CL5.1 were used as the database for MS spectra searching by Proteome Discoverer (v2.2), and a total of 3217 proteins were identified. The relative expression of proteins was calculated via normalization to the intensity of the TMT reporter. The protein expression differences between CT vs. the control group were calculated by Student’s *t*-test. The threshold of significant differentially expressed proteins (DEPs) between two groups was set as FC ≥ 1.5 or ≤ 0.67 and adjusted *p*-value < 0.05. There were 190 significantly up-regulated and 405 significantly down-regulated proteins in CT_34_ vs. control group. Similarly, there were 101 significantly up-regulated and 200 significantly down-regulated proteins in CT_72_ vs. the control group.

### Heterologous expression confirmation of CAP oxidation function of capO

Gene capO was amplified by PCR from the genomic DNA of *Sphingomonas* sp. CL5.1 using the primers 5′-GTCGACGGTATCGATTAAGGAGGTTTTCTAGTGCAAGATATTAGAACTAC-3′ and 5′-CAGGAATTCGATATCTCAGTGGCTTCTTCGGATCA-3′. The 50 μL PCR mixture system contained 25 μL of 2 × Phanta Max PCR Master Mix (Vazyme Biotech Co., Ltd., Nanjing, China), 2 μL each of forward and reverse primers, 1 μL of template DNA, and 20 μL of ddH_2_O. The PCR protocols were set as below: initial denaturation at 94 °C for 10 min, followed by 30 cycles of denaturation at 94 °C for 30 s, annealing at 60 °C for 30 s, and elongation at 72 °C for 1 min, with a final extension at 72 °C for 5 min. Then, the PCR product with the correct size was purified and confirmed by DNA sequencing. The recombinant vector pBBR1MCS-2 (pBBR) harboring the cloned capO (pBBR-capO) was constructed through homologous recombination using a seamless cloning kit (SparkJade Biotechnology Co., Ltd., Shandong, China). Finally, the constructed recombinant plasmid pBBR-capO was transformed into *Pseudomonas putida* KT2440 through electroporation according to the method established by Cho et al. [74]. Meanwhile, a recombinant *Pseudomonas putida* KT2440 carrying an empty pBBR was constructed as a control strain. The overnight cultures of recombinant *Pseudomonas putida* KT2440 strains were inoculated to MSM containing 100 mg/L CAP for the CAP biotransformation test.

## Supplementary Information

**Additional file 1: Table S1.** The taxonomy, quality, and genome size of 18 MAGs. **Table S2.** The taxonomy, quality, and genomic information of seven isolated strains. **Table S3.** The information of proteins related to CAP biotransformation in *Sphingomonas* sp. CL5.1 revealed by proteomics.

**Additional file 2: Figure S1.** The antimicrobial activity of the culture medium collected at various CAP biodegradation stages. **Figure S2.** Genomic characteristics of the seven isolated strains. **Figure S3.** The integrated analysis pipeline for multi-omics datasets. **Figure S4.** The KEGG enrichment analysis of DEGs and correlation analysis of DEGs with CAP as well as its metabolites. **Figure S5.** The HPLC-QTOF-MS chromatograms of main metabolites produced by isolated CAP-degrading strains including *Sphingomonas* sp. CL5.1, *Caballeronia* sp. PC1, *Cupriavidus* sp. CLC6. **Figure S6.** CAP biotransformation capacity of *Pseudomonas* putida KT2440 harboring gene *capO* which was cloned from *Sphingomonas* sp. CL5.1. **Figure S7.** The chiral chromatograms of four CAP stereoisomers standards and RS-CAP produced by *Caballeronia* sp. PC1. **Figure S8.** The temporal expression of genes involved in the glycine cleavage system of *Sphingomonas* sp. (MAG1). **Figure S9.** The dynamics of other CAP metabolites produced by isolated strains including *Sphingomonas* sp. CL5.1, *Caballeronia* sp. PC1, and *Cupriavidus* sp. CLC6, *Pigmentiphaga* sp. CLB6.2, *Chryseobacterium* sp. RCL7, *Labrys* sp. PNB5, and *Achromobacter* sp. CLB4. **Figure S10.** Comparison of gene clusters involved in the cleavage of a benzene ring in genomes of *Cupriavidus* sp. CLC6, *Sphingomonas* sp. CL5.1, *Caballeronia* sp. PC1, and *Bosea* sp. MAG8. **Figure S11.** The co-metabolism of CAP, PNB, and DCA by isolated strains including *Sphingomonas* sp. CL5.1, *Caballeronia* sp. PC1, and *Cupriavidus* sp. CLC6, *Pigmentiphaga* sp. CLB6.2, *Chryseobacterium* sp. RCL7, *Labrys* sp. PNB5, and *Achromobacter* sp. CLB4. **Figure S12.** The biotransformation of CAP by isolated strains in co-culture. **Figure S13.** The mineralization of CAP by *Sphingomonas* sp. CL 5.1 and its co-culture with other strains.

**Additional file 3.** The MS/MS spectra of CAP and its products determined by HPLC-QTOF-MS.

## Data Availability

Genomes of 7 isolated strains were deposited to NCBI with project IDs of PRJNA832945, PRJNA792803, and PRJNA576328. Forty-eight metagenomic datasets and 48 metatranscriptomic datasets were deposited to PRJNA833727 of the NCBI SRA database.
