# Supplementary material for: Deciphering chloramphenicol biotransformation mechanisms and microbial interactions via integrated multi-omics and cultivation-dependent approaches
Source: Microbiome. 2022 Oct 24;10:180. doi: 10.1186/s40168-022-01361-5 (PMC9590159; doi:10.1186/s40168-022-01361-5)
Supplement: Supplementary file 3 — Additional file 2: Figure S1. The antimicrobial activity of the culture medium collected at various CAP biodegradation stages. Figure S2. Genomic characteristics of the seven isolated strains. Figure S3. The integrated analysis pipeline for multi-omics datasets. Figure S4. The KEGG enrichment analysis of DEGs and correlation analysis of DEGs with CAP as well as its metabolites. Figure S5. The HPLC-QTOF-MS chromatograms of main metabolites produced by isolated CAP-degrading strains including Sphingomonas sp. CL5.1, Caballeronia sp. PC1, Cupriavidus sp. CLC6. Figure S6. CAP biotransformation capacity of Pseudomonas putida KT2440 harboring gene capO which was cloned from Sphingomonas sp. CL5.1. Figure S7. The chiral chromatograms of four CAP stereoisomers standards and RS-CAP produced by Caballeronia sp. PC1. Figure S8. The temporal expression of genes involved in the glycine cleavage system of Sphingomonas sp. (MAG1). Figure S9. The dynamics of other CAP metabolites produced by isolated strains including Sphingomonas sp. CL5.1, Caballeronia sp. PC1, and Cupriavidus sp. CLC6, Pigmentiphaga sp. CLB6.2, Chryseobacterium sp. RCL7, Labrys sp. PNB5, and Achromobacter sp. CLB4. Figure S10. Comparison of gene clusters involved in the cleavage of a benzene ring in genomes of Cupriavidus sp. CLC6, Sphingomonas sp. CL5.1, Caballeronia sp. PC1, and Bosea sp. MAG8. Figure S11. The co-metabolism of CAP, PNB, and DCA by isolated strains including Sphingomonas sp. CL5.1, Caballeronia sp. PC1, and Cupriavidus sp. CLC6, Pigmentiphaga sp. CLB6.2, Chryseobacterium sp. RCL7, Labrys sp. PNB5, and Achromobacter sp. CLB4. Figure S12. The biotransformation of CAP by isolated strains in co-culture. Figure S13. The mineralization of CAP by Sphingomonas sp. CL 5.1 and its co-culture with other strains. [file 40168_2022_1361_MOESM2_ESM.pdf]

# Supplementary information for

## Deciphering chloramphenicol biotransformation mechanisms and microbial interactions via integrated multi-omics and cultivation-dependent approaches

## This file includes:

**Fig. S1.** The antimicrobial activity of the culture medium collected at various CAP biodegradation stages

**Fig. S2** Genomic characteristics of the seven isolated strains

**Fig. S3** The integrated analysis pipeline for multi-omics datasets

**Fig. S4** The KEGG enrichment analysis of DEGs and correlation analysis of DEGs with CAP as well as its metabolites

**Fig. S5** The HPLC-QTOF-MS chromatograms of main metabolites produced by isolated CAP-degrading strains including *Sphingomonas* sp. CL5.1, *Caballeronia* sp. PC1, *Cupriavidus* sp. CLC6

**Fig. S6** CAP biotransformation capacity of *Pseudomonas* putida KT2440 harboring gene *capO* which was cloned from *Sphingomonas* sp. CL5.1

**Fig. S7** The chiral chromatograms of four CAP stereoisomers standards and RS-CAP produced by *Caballeronia* sp. PC1

**Fig. S8** The temporal expression of genes involved in the glycine cleavage system of *Sphingomonas* sp. (MAG1)

**Fig. S9** The dynamics of other CAP metabolites produced by isolated strains including *Sphingomonas* sp. CL5.1, *Caballeronia* sp. PC1, and *Cupriavidus* sp. CLC6, *Pigmentiphaga* sp. CLB6.2, *Chryseobacterium* sp. RCL7, *Labrys* sp. PNB5, and *Achromobacter* sp. CLB4

**Fig. S10** Comparison of gene clusters involved in the cleavage of a benzene ring in genomes of *Cupriavidus* sp. CLC6, *Sphingomonas* sp. CL5.1, *Caballeronia* sp. PC1, and *Bosea* sp. MAG8

**Fig. S11** The co-metabolism of CAP, PNB, and DCA by isolated strains including *Sphingomonas* sp. CL5.1, *Caballeronia* sp. PC1, and *Cupriavidus* sp. CLC6, *Pigmentiphaga* sp. CLB6.2, *Chryseobacterium* sp. RCL7, *Labrys* sp. PNB5, and *Achromobacter* sp. CLB4

**Fig. S12** The biotransformation of CAP by isolated strains in co-culture

**Fig. S13** The mineralization of CAP by *Sphingomonas* sp. CL 5.1 and its co-culture with other strains

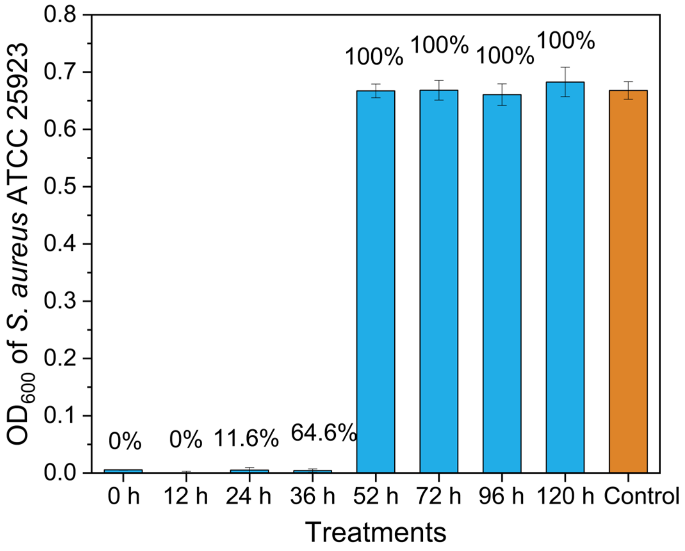


**Fig. S1. The antimicrobial activity of the culture medium collected at various CAP biodegradation stages.** The antimicrobial activity of CAP and its product residues in the culture medium is indicated by the growth inhibition on *Staphylococcus aureus* ATCC 25923. The removal efficiencies of CAP at an initial concentration of 120 mg/L in the collected culture medium samples are labeled at the top of the histogram. The Control was used to monitor the biomass of *Staphylococcus aureus* ATCC 25923 in the culture medium in the absence of CAP.

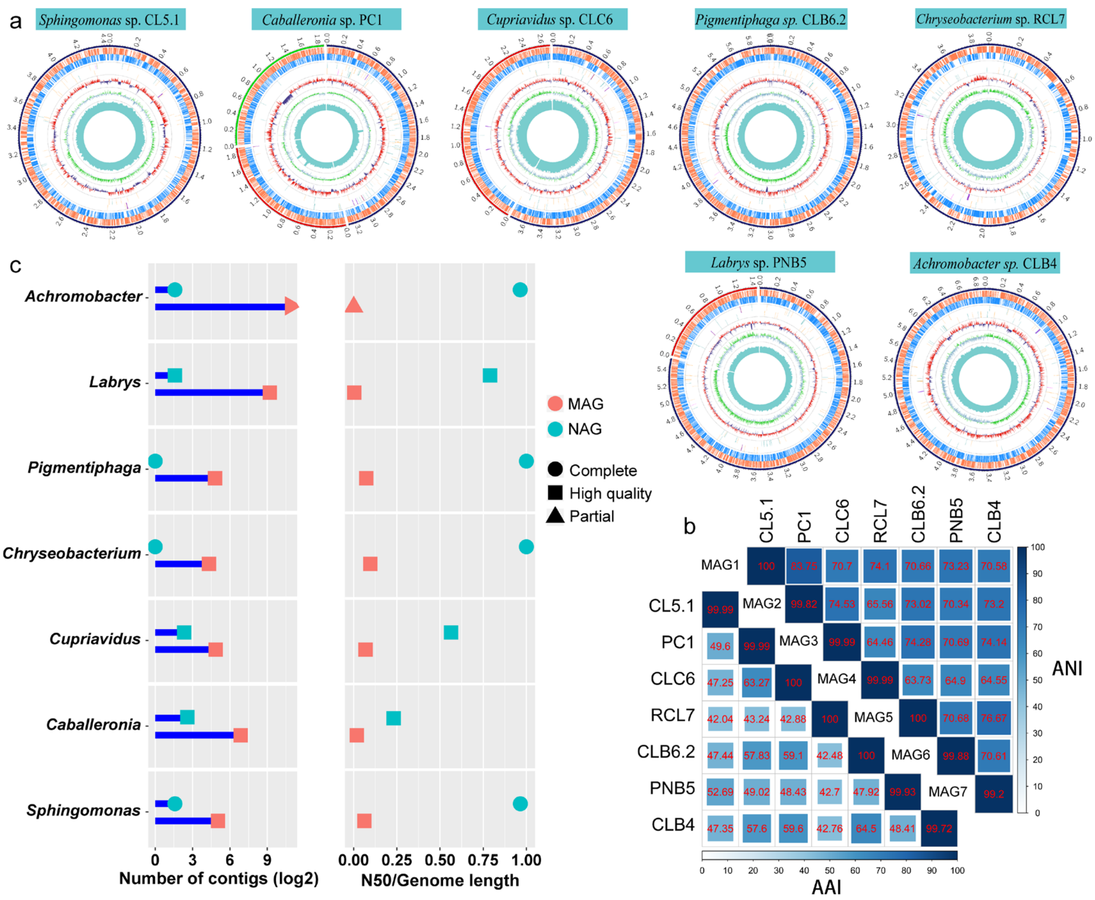


**Fig. S2. Genomic characteristics of the seven isolated strains. a.** Genomic features of chromosomal genomes. The concentric circles from outside to inside represent the following genomic features: 1st, CDS in sense sequence; 2nd, CDS in antisense sequence; 3rd, rRNA in the color of purple, tRNA in the color of orange; 4th, GC ratio; 5th, GC skew; and 6th, sequencing depth. **b.** The comparison of ANI and AAI similarity. **c.** The comparison of genomic quality between NAGs of isolates and MAGs recovered from metagenomes. Complete genome: only one circularized chromosomal contig with completeness > 99% and contamination < 2.5%. High quality genome: completeness > 90% and contamination < 2.5%. Partial genome: completeness < 90%.

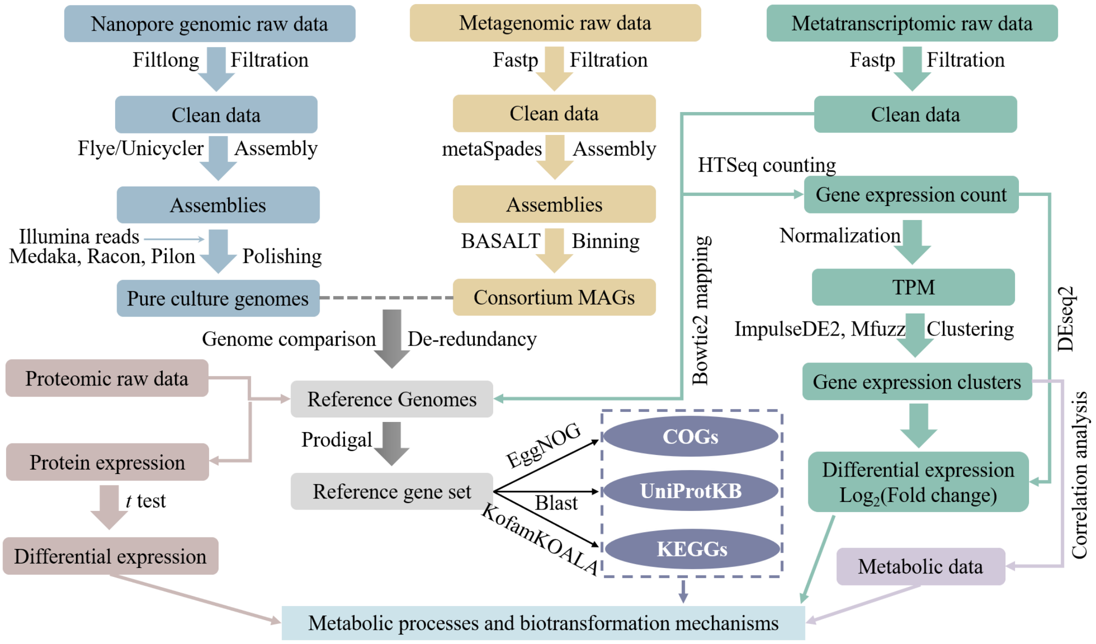


**Fig. S3. The integrated analysis pipeline for multi-omics datasets**

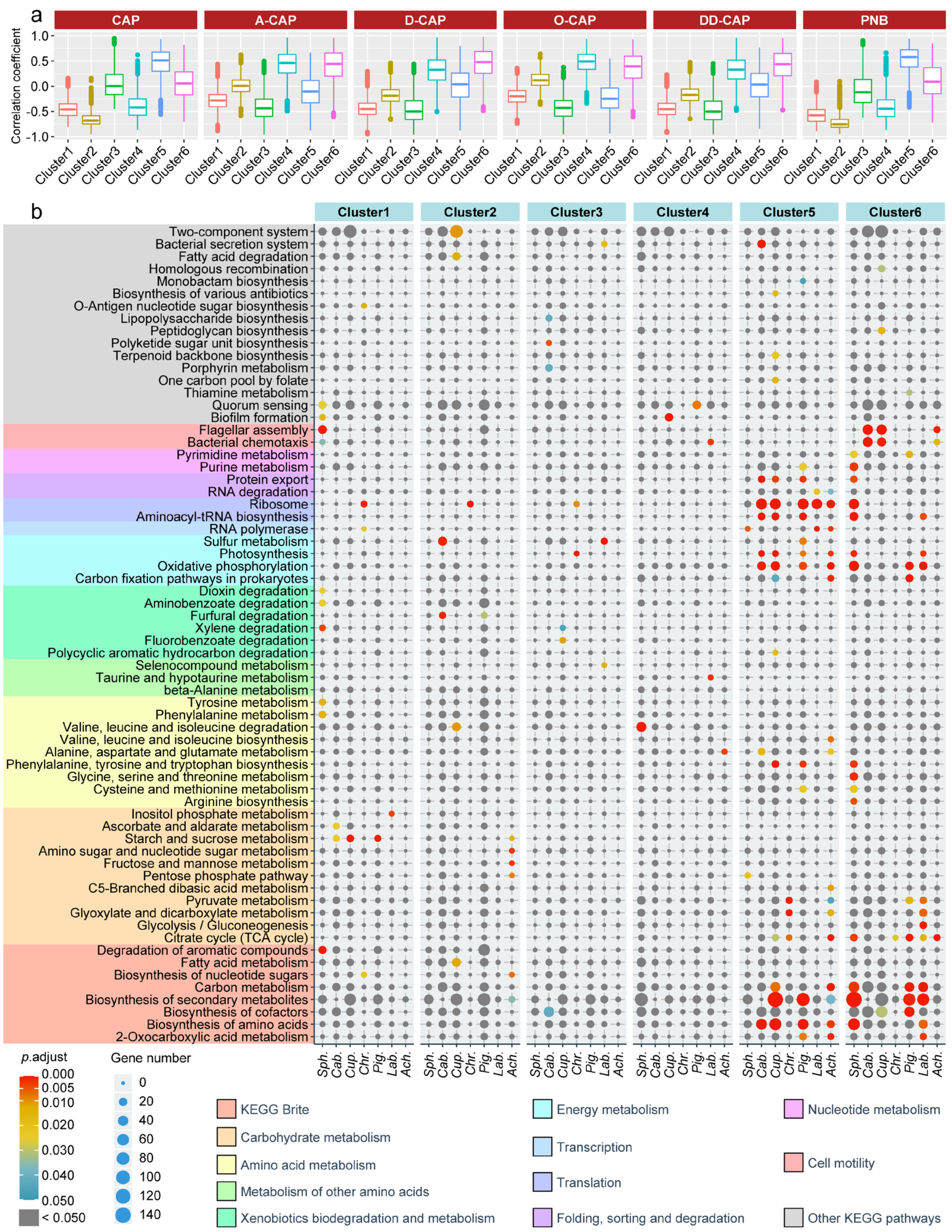


**Fig. S4. The KEGG enrichment analysis of DEGs and correlation analysis of DEGs with CAP as well as its metabolites. a.** The correlation analysis between DEGs and CAP as well as its metabolites. **b.** The enriched KEGG terms in each core species of consortium CL including *Sphingomonas* sp. (*Sph.*), *Caballeronia* sp. (*Cab.*), *Cupriavidus* sp. (*Cup.*), *Chryseobacterium* sp. (*Chr.*), *Pigmentiphaga* sp. (*Pig.*), *Labrys* sp. (*Lab.*), and *Achromobacter* sp. (*Ach.*).

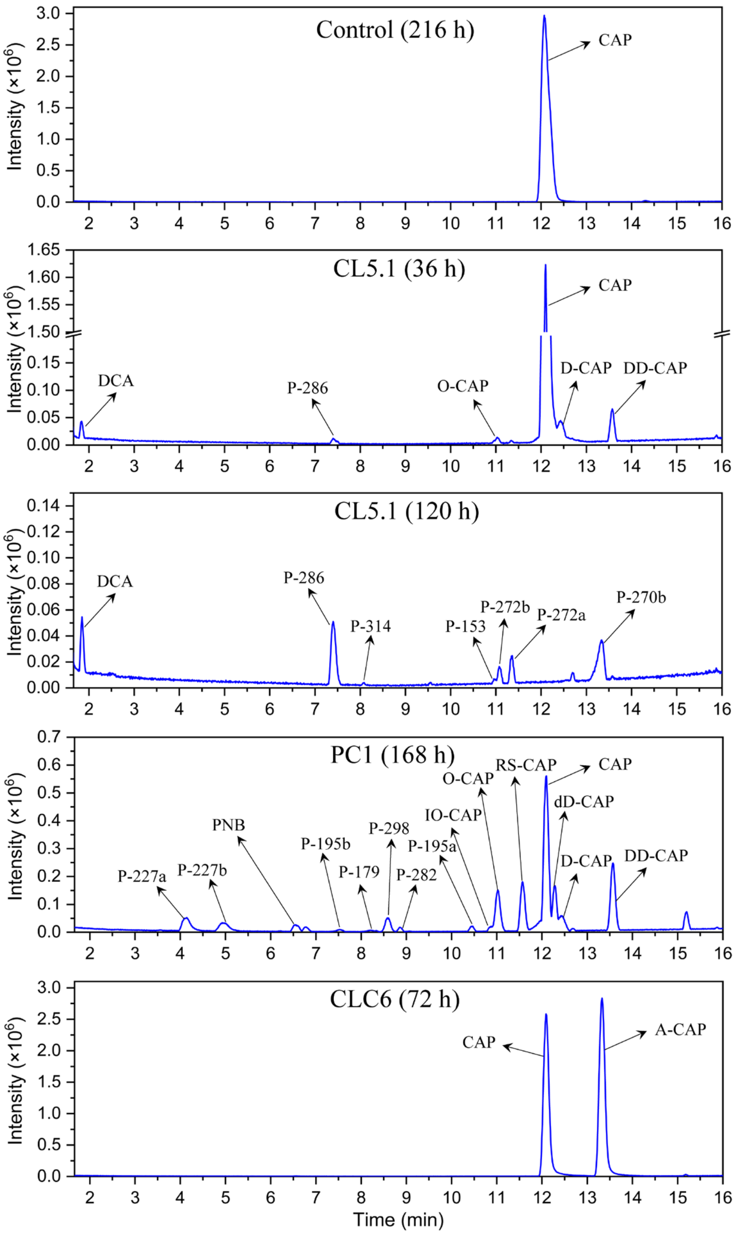


**Fig. S5. The HPLC-QTOF-MS chromatograms of main metabolites produced by isolated CAP-degrading strains including *Sphingomonas* sp. CL5.1, *Caballeronia* sp. PC1, *Cupriavidus* sp. CLC6.**

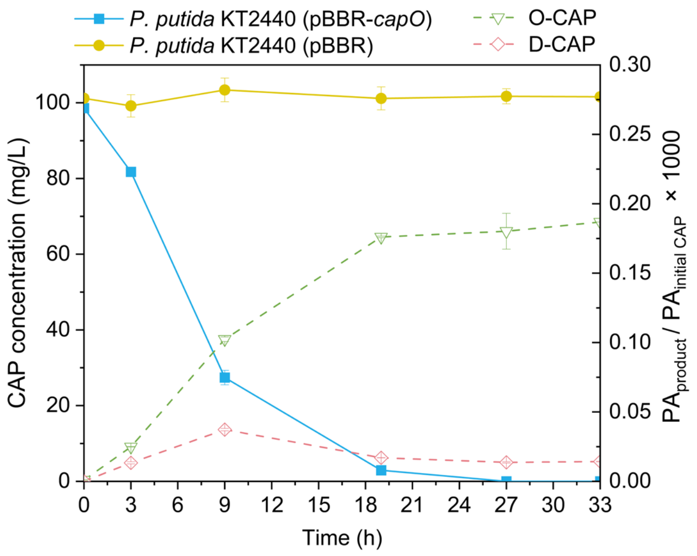


**Fig. S6. CAP biotransformation capacity of *Pseudomonas* putida KT2440 harboring gene *capO* which was cloned from *Sphingomonas* sp. CL5.1.** The blue and orange solid lines represent CAP concentrations in the culture medium inoculated with *Pseudomonas putida* KT2440 (pBBR-*capO*) carrying *capO* and *Pseudomonas putida* KT2440 (pBBR) containing an empty plasmid, respectively. O-CAP and D-CAP were semi-quantified according to the proportion of their peak areas (PA) to the peak area of CAP at the initial concentration of 100 mg/L. Data are presented as mean values ± SD (n = 3).

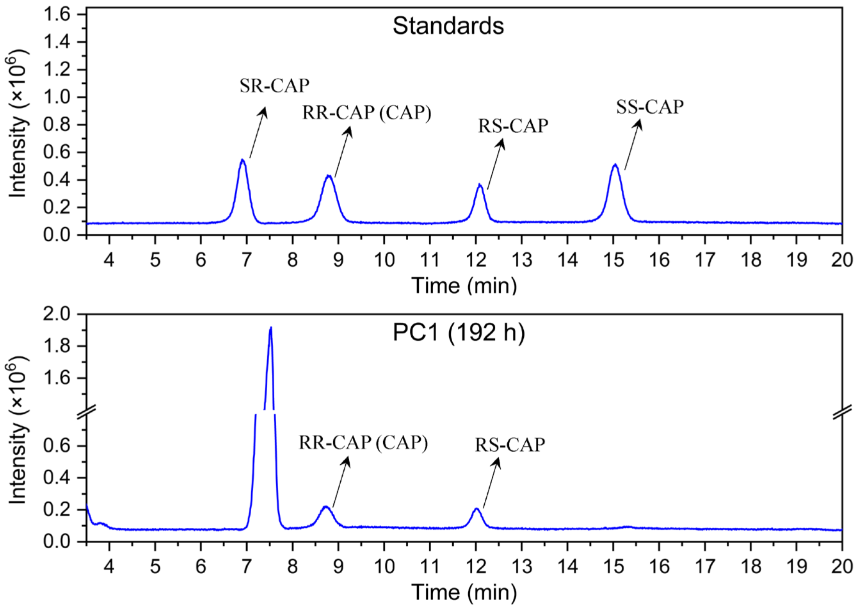


**Fig. S7. The chiral chromatograms of four CAP stereoisomers standards and RS-CAP produced by *Caballeronia* sp. PC1.** The detection was conducted using HPLC-QTOF-MS with a Chiralpak AGP column.

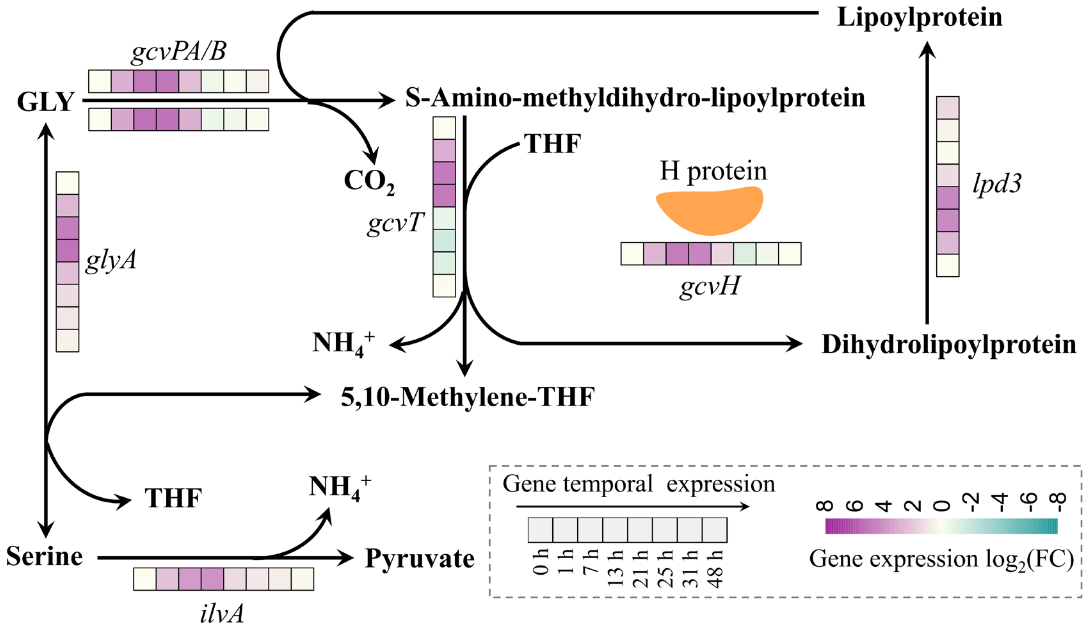


**Fig. S8. The temporal expression of genes involved in the glycine cleavage system of *Sphingomonas* sp. (MAG1).** The little squares in various colors indicated the gene expression difference at different sampling times compared to that at 0 h (before CAP dosing). THF: Tetrahydrofolate.

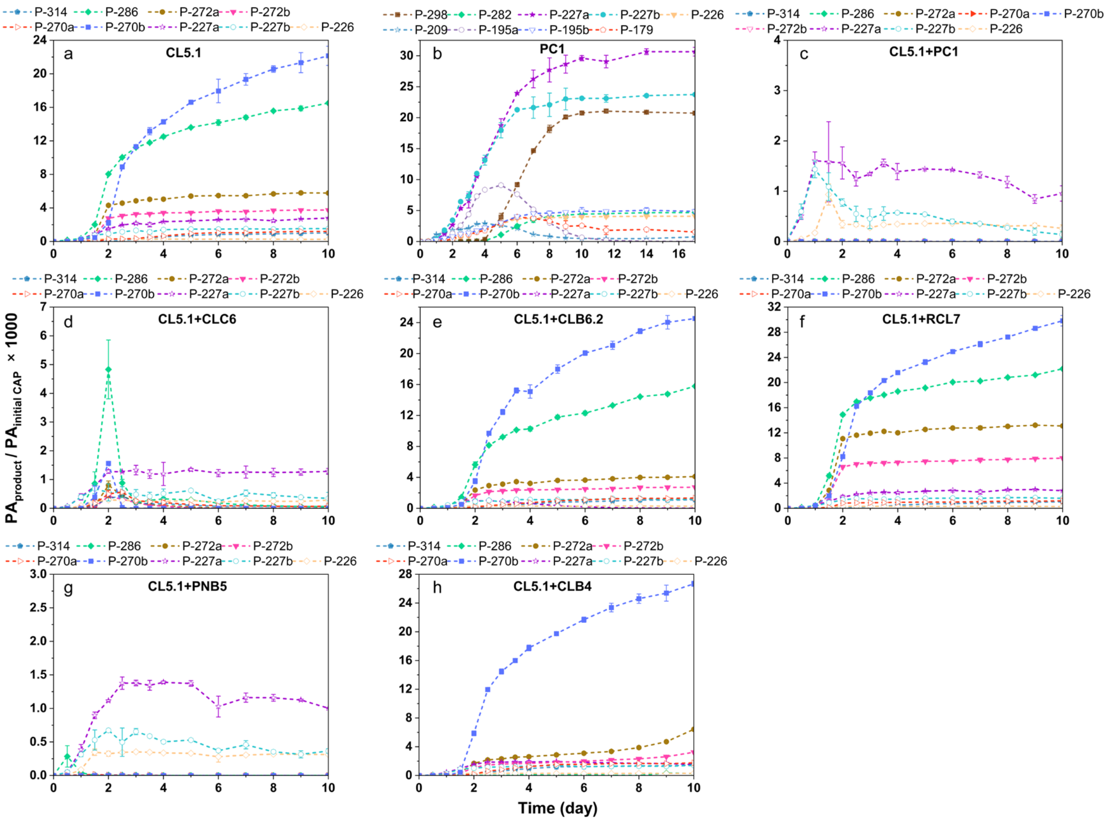


**Fig. S9. The dynamics of other CAP metabolites produced by isolated strains including *Sphingomonas* sp. CL5.1, *Caballeronia* sp. PC1, and *Cupriavidus* sp. CLC6, *Pigmentiphaga* sp. CLB6.2, *Chryseobacterium* sp. RCL7, *Labrys* sp. PNB5, and *Achromobacter* sp. CLB4. a-b,** The dynamics of CAP metabolites produced by the axenic culture. **c-h,** The dynamics of CAP metabolites produced by mixed strains. Metabolites were semi-quantified according to the proportion of their peak areas (PA) to the peak area of CAP at the initial concentration of 120 mg/L. Data are presented as mean values ± SD (n = 3).

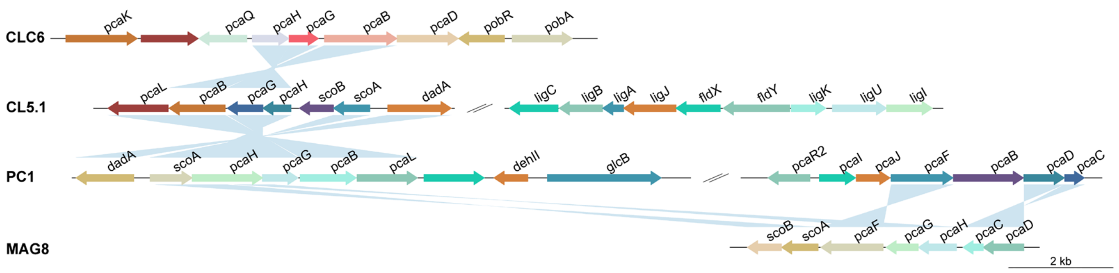


**Fig. S10. Comparison of gene clusters involved in the cleavage of a benzene ring in genomes of *Cupriavidus* sp. CLC6, *Sphingomonas* sp. CL5.1, *Caballeronia* sp. PC1, and *Bosea* sp. MAG8.**

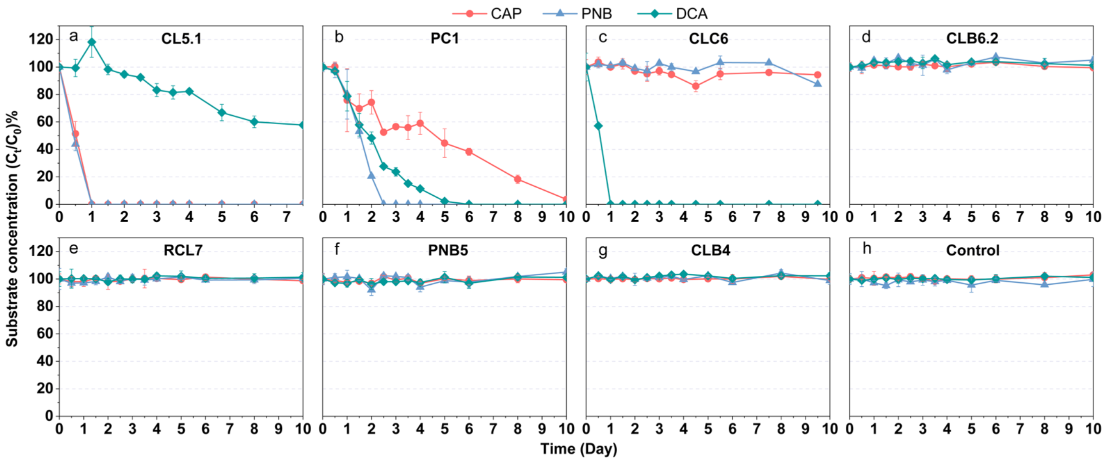


**Fig. S11. The co-metabolism of CAP, PNB, and DCA by isolated strains including *Sphingomonas* sp. CL5.1, *Caballeronia* sp. PC1, and *Cupriavidus* sp. CLC6, *Pigmentiphaga* sp. CLB6.2, *Chryseobacterium* sp. RCL7, *Labrys* sp. PNB5, and *Achromobacter* sp. CLB4.** The initial concentrations of CAP, PNB, and DCA were all 50 mg/L. Control indicates the control group without bacteria inoculation. Data are presented as mean values ± SD (n = 3).

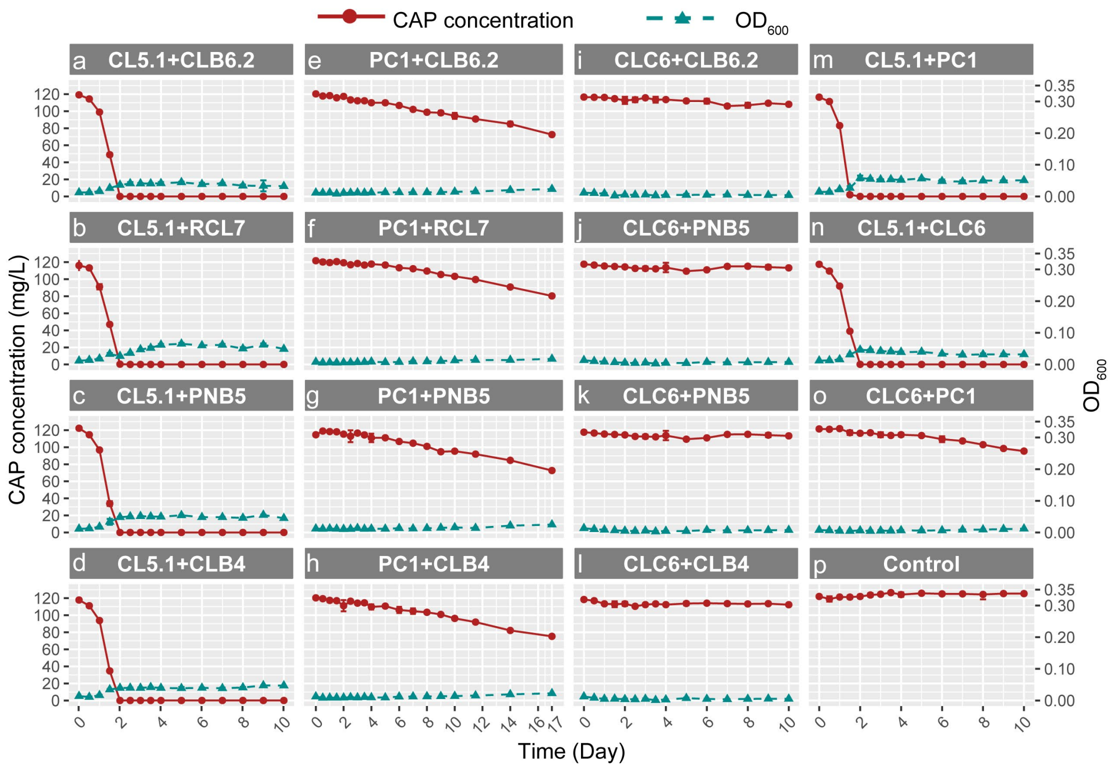


**Fig. S12. The biotransformation of CAP by isolated strains in co-culture.** The biodegradation of CAP by co-culture of seven isolated strains including *Sphingomonas* sp. CL5.1, *Caballeronia* sp. PC1, *Cupriavidus* sp. CLC6, *Pigmentiphaga* sp. CLB6.2, *Chryseobacterium* sp. RCL7, *Labrys* sp. PNB5, and *Achromobacter* sp. CLB4 was conducted with the presence of 30 mg/L ammonium chloride. Control indicates the control group without bacteria inoculation. Data are presented as mean values ± SD (n = 3).

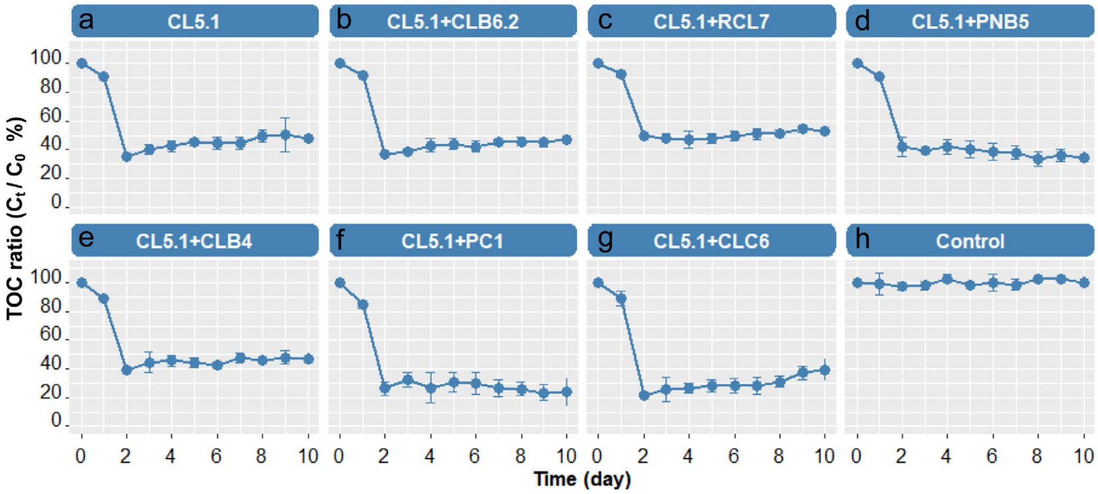


**Fig. S13. The mineralization of CAP by *Sphingomonas* sp. CL 5.1 and its co-culture with other strains.** Control indicates the control group without bacteria inoculation. Data are presented as mean values ± SD (n = 3).
