# Supplementary material for: Deciphering chloramphenicol biotransformation mechanisms and microbial interactions via integrated multi-omics and cultivation-dependent approaches
Source: Microbiome. 2022 Oct 24;10:180. doi: 10.1186/s40168-022-01361-5 (PMC9590159; doi:10.1186/s40168-022-01361-5)
Supplement: Supplementary file 4 — Additional file 3. The MS/MS spectra of CAP and its products determined by HPLC-QTOF-MS. [file 40168_2022_1361_MOESM3_ESM.pptx]

## Slide 1
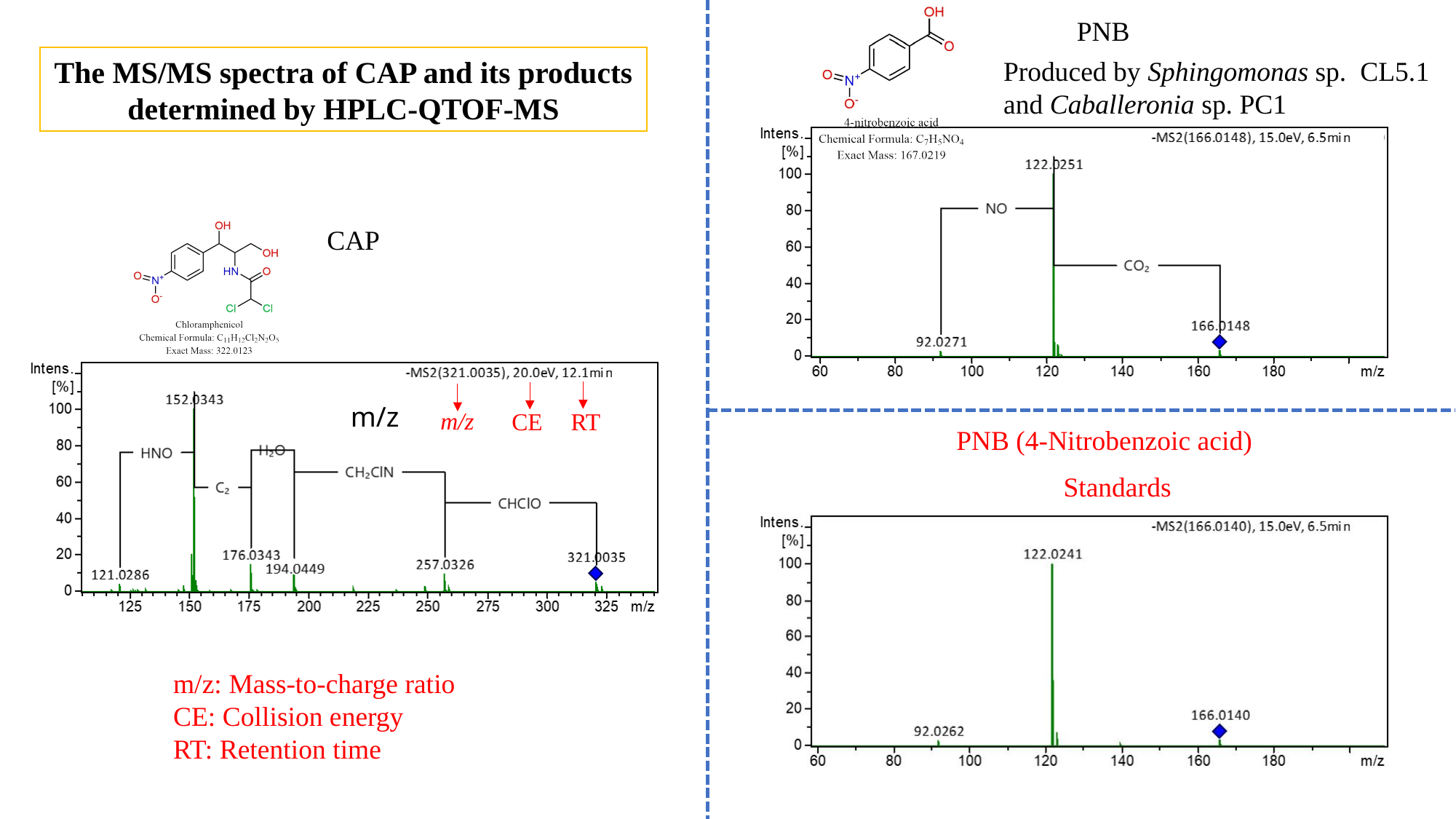

PNB
The MS/MS spectra of CAP and its products determined by HPLC-QTOF-MS
Produced by Sphingomonas sp. CL5.1 and Caballeronia sp. PC1
CAP
m/z
m/z
CE
RT
PNB (4-Nitrobenzoic acid)
Standards
m/z: Mass-to-charge ratio
CE: Collision energy
RT: Retention time

## Slide 2
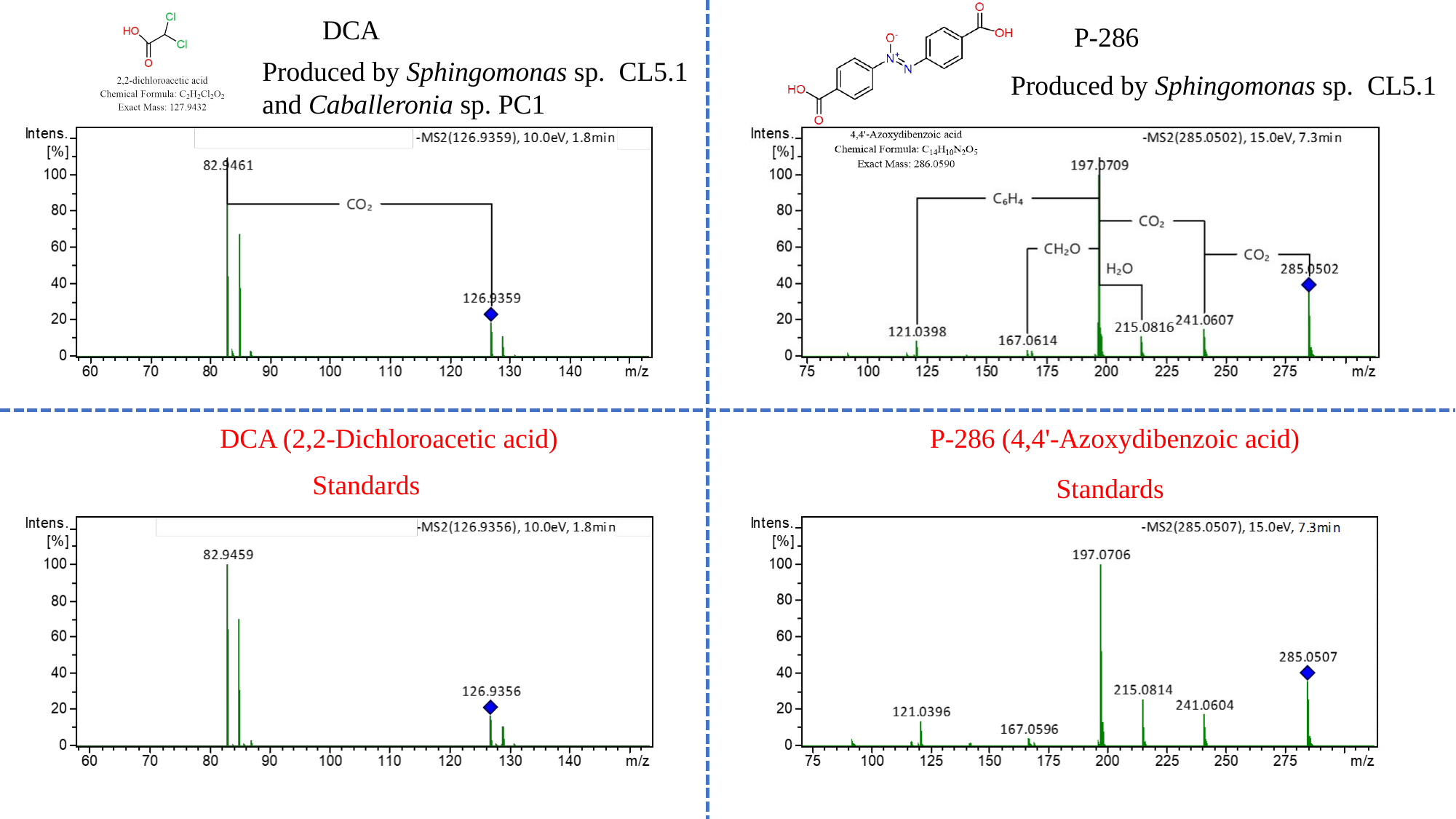

DCA
P-286
Produced by Sphingomonas sp. CL5.1 and Caballeronia sp. PC1
Produced by Sphingomonas sp. CL5.1
DCA (2,2-Dichloroacetic acid)
P-286 (4,4'-Azoxydibenzoic acid)
Standards
Standards

## Slide 3
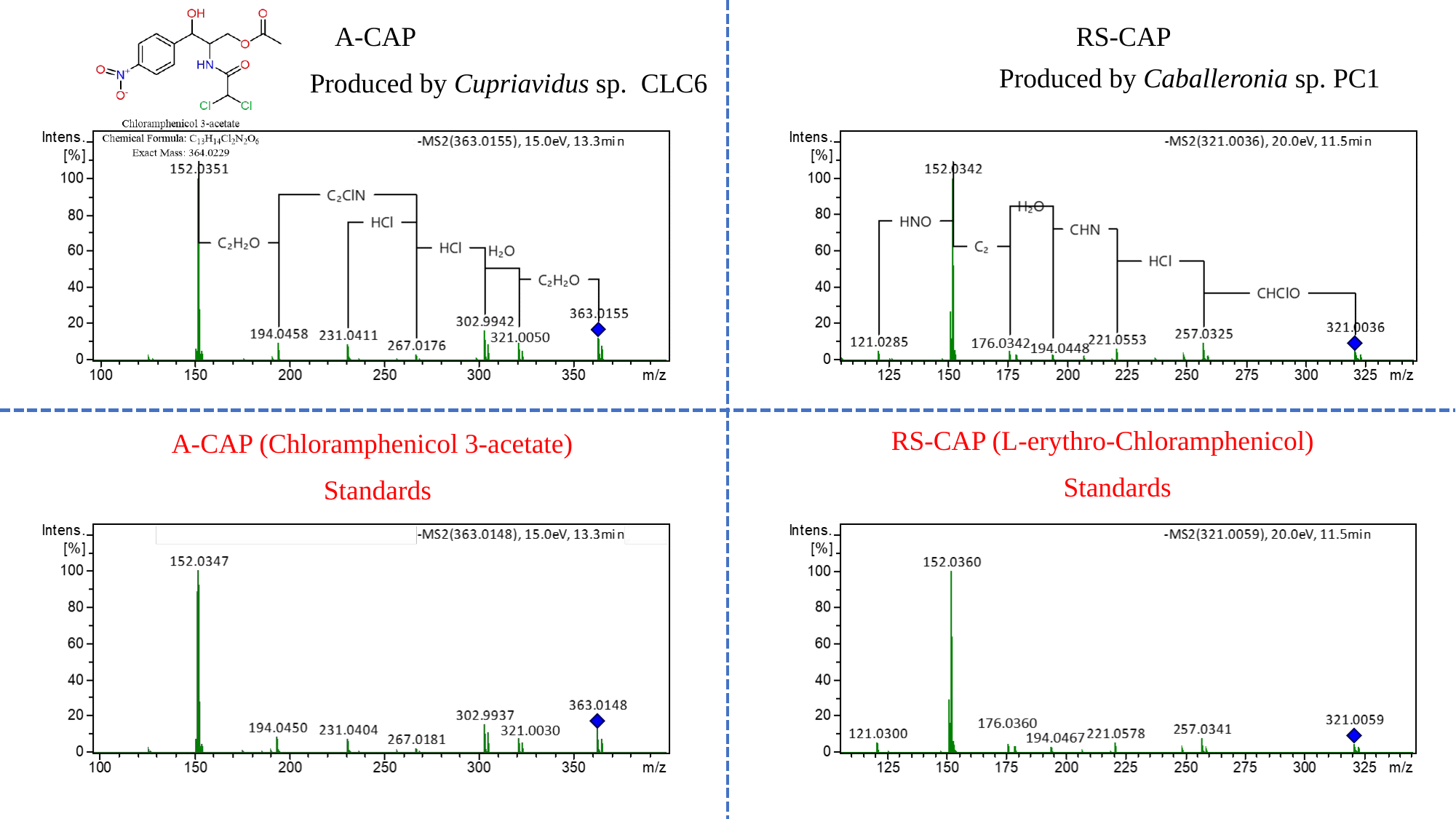

A-CAP
RS-CAP
Produced by Caballeronia sp. PC1
Produced by Cupriavidus sp. CLC6
RS-CAP (L-erythro-Chloramphenicol)
A-CAP (Chloramphenicol 3-acetate)
Standards
Standards

## Slide 4
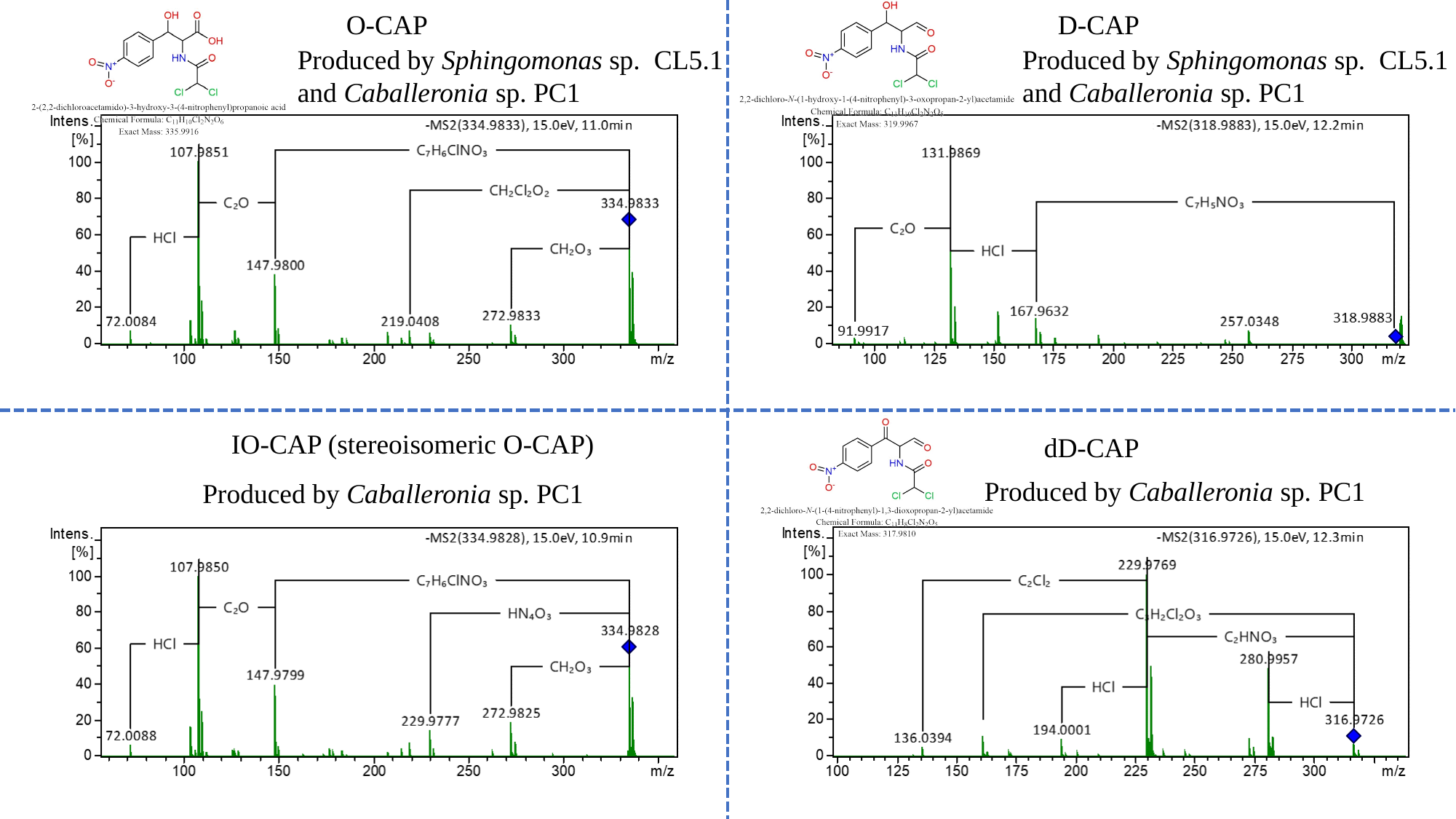

D-CAP
O-CAP
Produced by Sphingomonas sp. CL5.1 and Caballeronia sp. PC1
Produced by Sphingomonas sp. CL5.1 and Caballeronia sp. PC1
IO-CAP (stereoisomeric O-CAP)
dD-CAP
Produced by Caballeronia sp. PC1
Produced by Caballeronia sp. PC1

## Slide 5
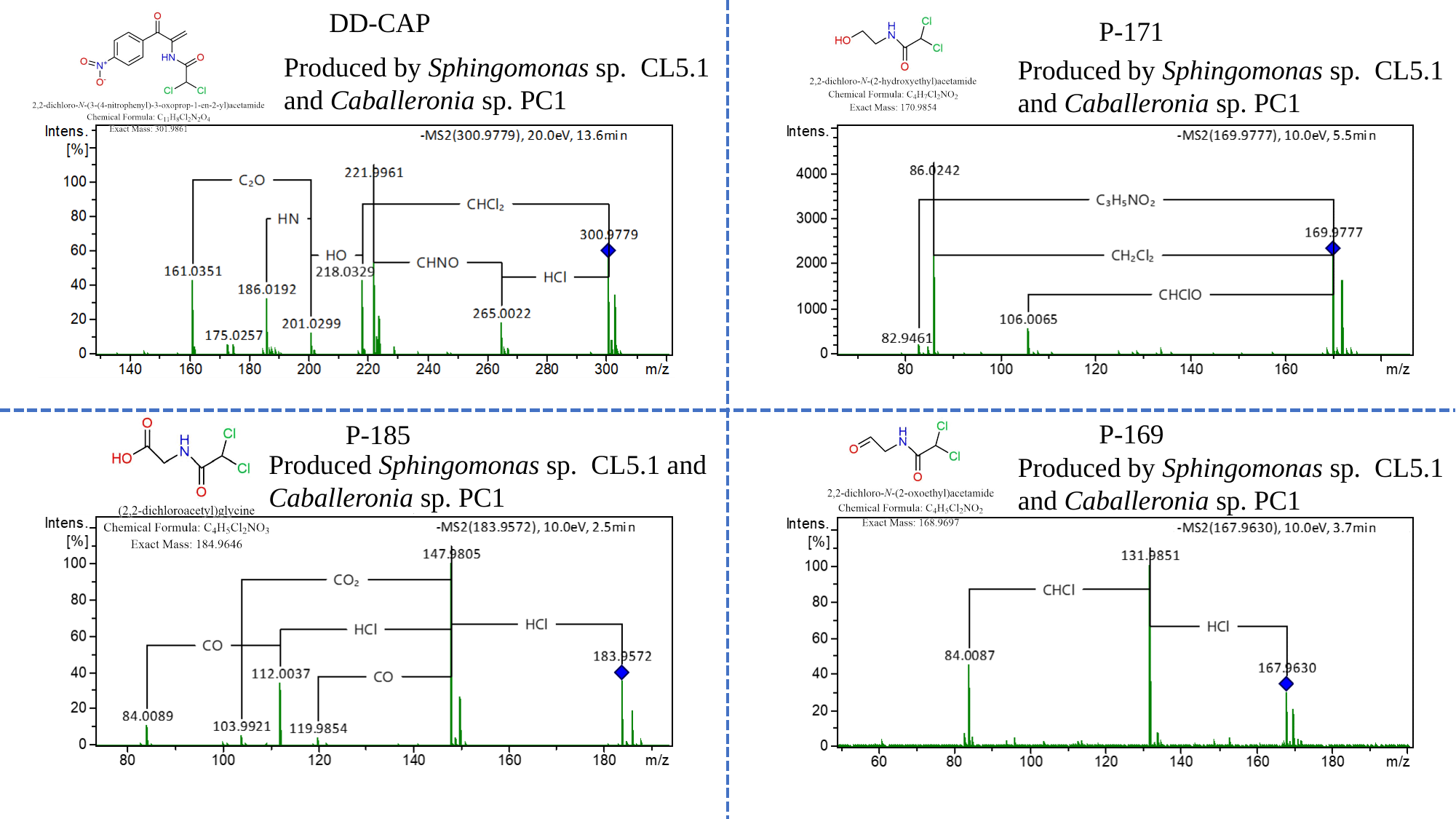

DD-CAP
P-171
Produced by Sphingomonas sp. CL5.1 and Caballeronia sp. PC1
Produced by Sphingomonas sp. CL5.1 and Caballeronia sp. PC1
P-169
P-185
Produced Sphingomonas sp. CL5.1 and Caballeronia sp. PC1
Produced by Sphingomonas sp. CL5.1 and Caballeronia sp. PC1

## Slide 6
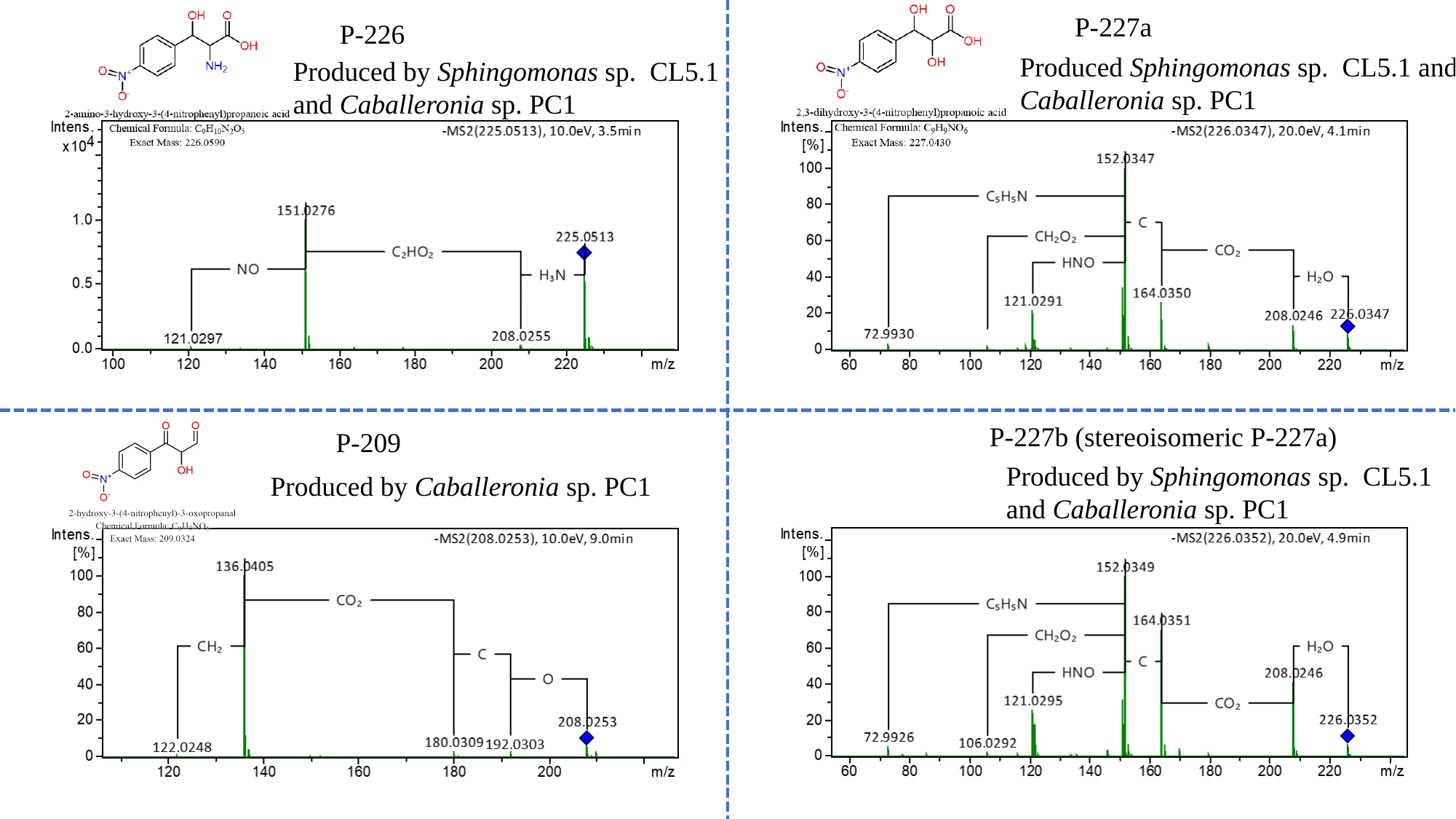

P-227a
P-226
Produced Sphingomonas sp. CL5.1 and Caballeronia sp. PC1
Produced by Sphingomonas sp. CL5.1 and Caballeronia sp. PC1
P-227b (stereoisomeric P-227a)
P-209
Produced by Sphingomonas sp. CL5.1 and Caballeronia sp. PC1
Produced by Caballeronia sp. PC1

## Slide 7
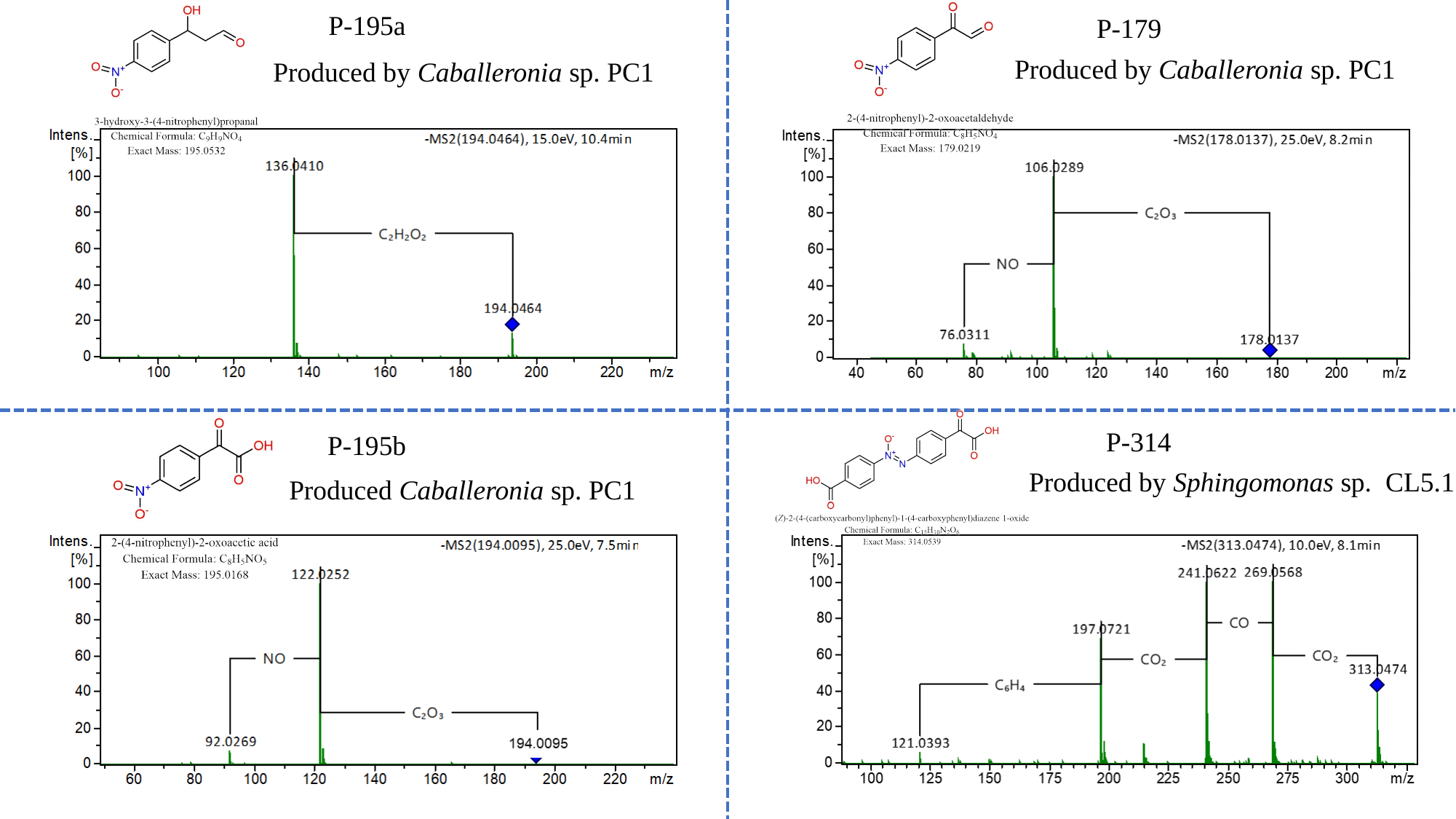

P-195a
P-179
Produced by Caballeronia sp. PC1
Produced by Caballeronia sp. PC1
P-314
P-195b
Produced by Sphingomonas sp. CL5.1
Produced Caballeronia sp. PC1

## Slide 8
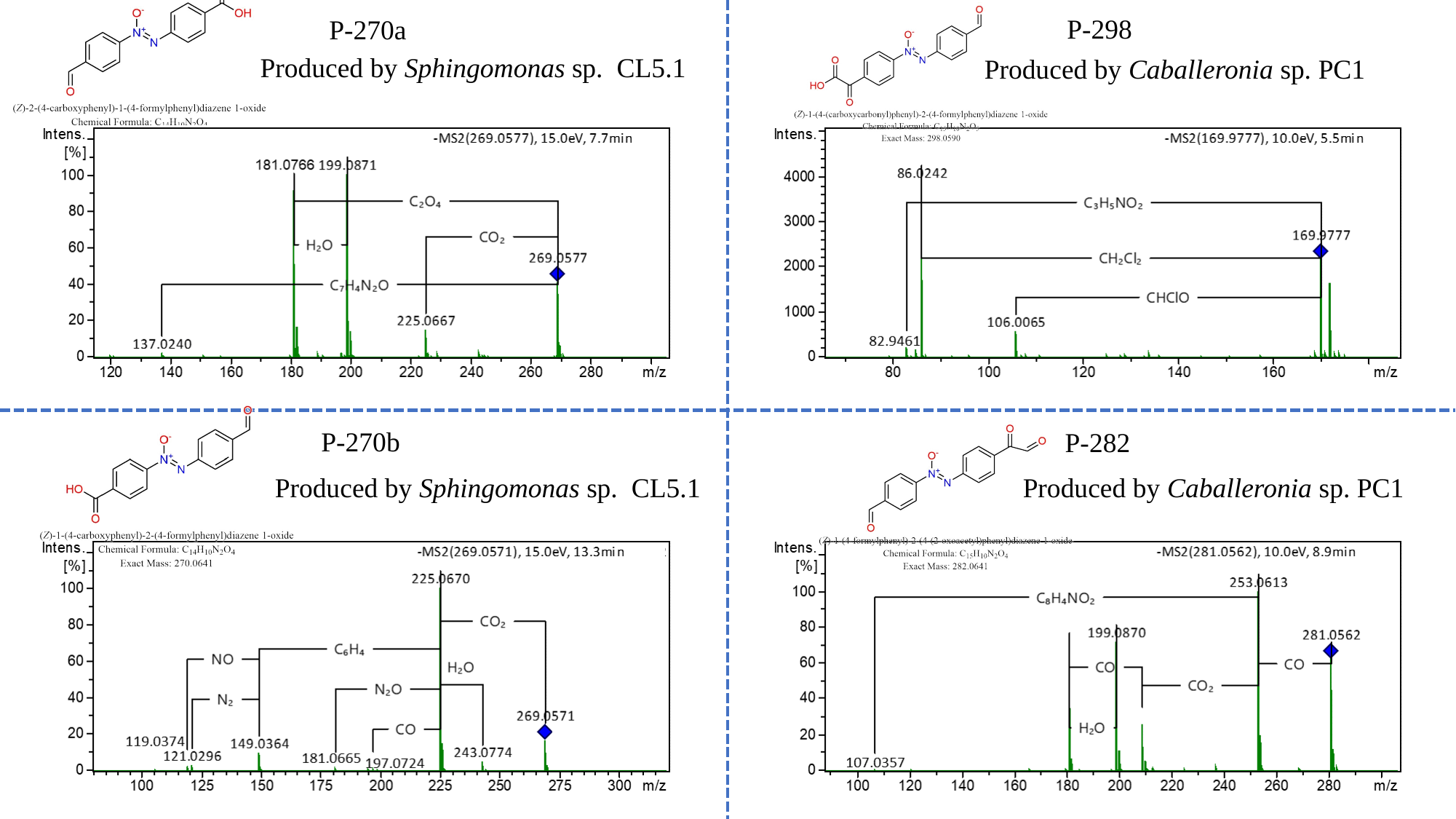

P-298
P-270a
Produced by Sphingomonas sp. CL5.1
Produced by Caballeronia sp. PC1
P-270b
P-282
Produced by Sphingomonas sp. CL5.1
Produced by Caballeronia sp. PC1

## Slide 9
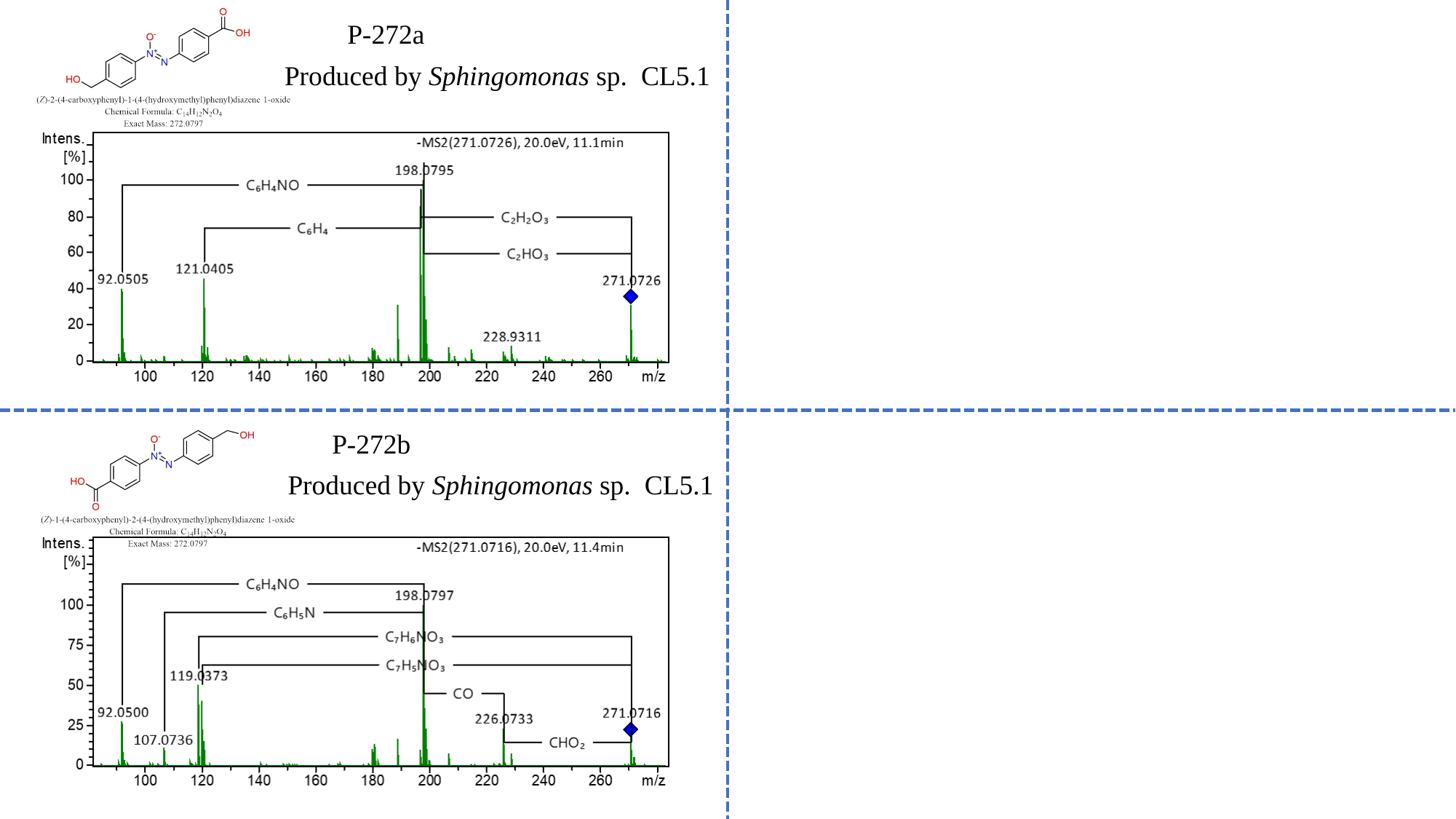

P-272a
Produced by Sphingomonas sp. CL5.1
P-272b
Produced by Sphingomonas sp. CL5.1
